# Metabolic and cardiovascular benefits and risks of 4-hydroxy guanabenz hydrochloride: α_2_-adrenoceptor and trace amine-associated receptor 1 ligand

**DOI:** 10.1007/s43440-023-00518-9

**Published:** 2023-08-25

**Authors:** Magdalena Kotańska, Monika Marcinkowska, Kamil J. Kuder, Maria Walczak, Marek Bednarski, Agata Siwek, Marcin Kołaczkowski

**Affiliations:** 1 Department of Pharmacological Screening, Faculty of Pharmacy, Jagiellonian University Medical College, Kraków, Poland; 2 Department of Medicinal Chemistry, Faculty of Pharmacy, Jagiellonian University Medical College, Kraków, Poland; 3 Department of Technology and Biotechnology of Drugs, Faculty of Pharmacy, Jagiellonian University Medical College, Kraków, Poland; 4 Chair and Department of Toxicology, Faculty of Pharmacy, Jagiellonian University Medical College, Kraków, Poland; 5 Department of Pharmacobiology, Faculty of Pharmacy, Jagiellonian University Medical College, Kraków, Poland; 6 Adamed Pharma Ltd, Czosnów, Poland

**Keywords:** 4-Hydroxy guanabenz hydrochloride, Guanabenz, α_2_-adrenoceptor partial agonist, Trace amine-associated receptor 1, Obesity, Metabolic disturbances

## Abstract

**Background:**

α_2_-adrenoceptor ligands have been investigated as potential therapeutic agents for the treatment of obesity. Our previous studies have shown that guanabenz reduces the body weight of obese rats, presumably through its anorectic action. This demonstrates an additional beneficial effect on selected metabolic parameters, including glucose levels. The purpose of this present research was to determine the activity of guanabenz's metabolite—4-hydroxy guanabenz hydrochloride (4-OH-Guanabenz).

**Methods:**

We performed in silico analyses, involving molecular docking to targets of specific interest as well as other potential biological targets. In vitro investigations were conducted to assess the selectivity profile of 4-OH-Guanabenz binding to α-adrenoceptors, along with intrinsic activity studies involving α_2_-adrenoceptors and trace amine-associated receptor 1 (TAAR_1_). Additionally, the effects of 4-OH-Guanabenz on the body weight of rats and selected metabolic parameters were evaluated using the diet-induced obesity model. Basic safety and pharmacokinetic parameters were also examined.

**Results:**

4-OH-guanabenz is a partial agonist of α_2A_-adrenoceptor. The calculated EC_50_ value for it is 316.3 nM. It shows weak agonistic activity at TAAR_1_ too. The EC_50_ value for 4-OH-Guanabenz calculated after computer simulation is 330.6 µM. Its primary mode of action is peripheral. The penetration of 4-OH-Guanabenz into the brain is fast (*t*_max_ = 15 min), however, with a low maximum concentration of 64.5 ng/g. 4-OH-Guanabenz administered *ip* at a dose of 5 mg/kg b.w. to rats fed a high-fat diet causes a significant decrease in body weight (approximately 14.8% compared to the baseline weight before treatment), reduces the number of calories consumed by rats, and decreases plasma glucose and triglyceride levels.

**Conclusions:**

The precise sequence of molecular events within the organism, linking the impact of 4-OH-Guanabenz on α_2A_-adrenoceptor and TAAR_1_ with weight reduction and the amelioration of metabolic disturbances, remains an unresolved matter necessitating further investigation. Undoubtedly, the fact that 4-OH-Guanabenz is a metabolite of a well-known drug has considerable importance, which is beneficial from an economic point of view and towards its further development as a drug candidate.

**Supplementary Information:**

The online version contains supplementary material available at 10.1007/s43440-023-00518-9.

## Introduction

Catecholamines exert an influence on lipolysis through α_2_- and β-adrenoceptors [1–3]. α_2_- and β_3_-Adrenoceptors coexist within the same fat cell [2, 4]. Stimulation of α_2_-adrenoceptors results in the inhibition, whereas stimulation of β-adrenoceptors leads to the enhancement of lipolysis [1–4]. Therefore, the ratio of functional α_2_- and β_3_-adrenoceptors present in subcutaneous fat cells seems to dictate the activation of fat storage or release in response to catecholamines [4]. It is postulated that the fatty subcutaneous tissue in obese individuals is resistant to catecholamines [4]. In obese patients, a notable decrease in the expression and function of β-adrenoceptors within subcutaneous fat cells has been documented, juxtaposed with an augmentation in α_2_-adrenoceptor stimulation [4]. This phenomenon potentially contributes to a reduction in the intensity of lipolysis and the excessive accumulation of triglycerides within adipocytes [1, 4].

By blocking presynaptic α_2_-adrenoceptors and inhibiting the release of catecholamines, α_2_-adrenoceptor antagonists may promote an increase in sympathetic nervous system activity. Additionally, these antagonists may augment thermogenesis and suppress lipolysis by obstructing α_2_-adrenoceptors in adipose tissue, where their natural stimulation would otherwise lead to lipolysis reduction [5]. Consequently, the application of α_2_-adrenoceptor antagonists holds the potential to induce weight reduction among obese individuals.

The idea to employ the α_2_-adrenoceptor ligands in the treatment of obesity has previously been suggested [5–7]. In literature, there are reports that the anorectic effects of some effective drugs, such as sibutramine or bupropion, are associated with their influence on α_2_-adrenoceptors [8, 9]. α_2_-Adrenoceptors have been associated with the control of blood pressure, insulin secretion, and adipocyte function [5, 10, 11]. Therefore, finding a drug that affects the activity of these receptors in a suitable manner would be useful for treating not only obesity but also metabolic syndrome.

In recent years, the scientific interest in guanabenz (an old sympatholytic drug [12]) has been growing again due to the discovery of its beneficial properties, exerted not only specifically in the circulatory system but in other indications too. It has been proven that this drug can exhibit neuroprotective effects [13–15] and antiangiogenic activity [16], and reduce liver toxicity induced by acetaminophen administration [17]. Interestingly, it was also discovered to elicit a weight-reducing effect in obese rats, presumably through anorectic action [18], showing an additional beneficial effect on selected metabolic parameters, such as glucose levels [18, 19].

An interesting and effective option in the development of new active compounds is to investigate the activity of metabolites of known drugs. This is because metabolites are usually less toxic and safer. Organisms have an intrinsic tendency to diminish the harm posed by their exposure to chemicals (e.g., by structural modification) and excretion of them [20]. In some cases, a metabolite can retain enough activity at the target receptor, so that it may significantly contribute to the in vivo pharmacological effect(s) [21].

4-Hydroxy guanabenz (4-OH-Guanabenz) is a metabolite of guanabenz, which also possesses an affinity for the α_2_-adrenoceptor [22]. However, to date, the studies involving this compound are limited. In this research work, we examined the selectivity profile of 4-OH-Guanabenz, performing its binding toward α_1_- and α_2_-adrenoceptors and intrinsic activity studies toward the α_2A_- and α_2B_-adrenoceptors. The 4-OH-Guanabenz proved to be a partial agonist of α_2A_-adrenoceptor; therefore, we examined its influence on the body weight of obese animals and on selected metabolic parameters. Basic safety and pharmacokinetic parameters were also determined. Moreover, to test the possible promiscuity of the ligand, we used the target prediction server to look for other possible biological targets and performed preliminary docking studies with targets of specific interest.

## Materials and methods

### Animals

The experiments were carried out on male Wistar rats (6 months old). Initial body weight was: 140–160 g obesity model or 190–220 g rats in which the obesity model was not induced. The animals were housed in pairs in plastic cages in constant temperature facilities exposed to a light–dark cycle; water and food were available ad libitum. The control and experimental groups consisted of six-to-eight animals each. All experiments were carried out according to the guidelines of the Jagiellonian University Animal Use and Care Committee and were approved for realization (Permissions No. 54/2012 and No. 128/2017).

For the pharmacokinetic study, a group of 56 adult male rats (Wistar, 220–250 g, 8-month-old) were used in the experiment. Animals were purchased from the Animal House of the Faculty of Pharmacy of Jagiellonian University Medical College, Krakow, Poland. During the habituation period, the groups of 4 rats were kept in a plastic cage at a controlled room temperature (22 ± 2 ℃), humidity (55 ± 10%), full-spectrum cold white light (350–400 lx), in 12 h light/dark cycles (the lights on at 7:00 am, and off at 19:00 pm), and had free access to standard laboratory pellet and tap water. Guanabenz and 4-OH-Guanabenz were dissolved in water. These were then given through an intraperitoneally injections (*ip*) at a dose of 0.3 mg/kg and 0.4 mg/kg, respectively. Blood samples were collected at 0 min (predose), 5 min, 15 min, 30 min, 60 min, 120 min, and 240 min after compound administration. Blood and brain samples were collected under general anesthesia induced by *ip* injections of 50 mg/kg ketamine plus 8 mg/kg xylazine. Blood samples were taken in heparinized tubes and immediately centrifuged at 3500 rpm for 10 min, and plasma was collected. Brain and plasma samples were immediately frozen at – 80 ℃ for LC/MS/MS analysis. All experimental procedures were carried out according to EU Directive 2010/63/EU and approved by the I Local Ethics Committee for Animal Experiments of the Jagiellonian University in Krakow, Poland (No. 94/2016).

### Drugs, chemical reagents, and other materials

**Fig. 1:**
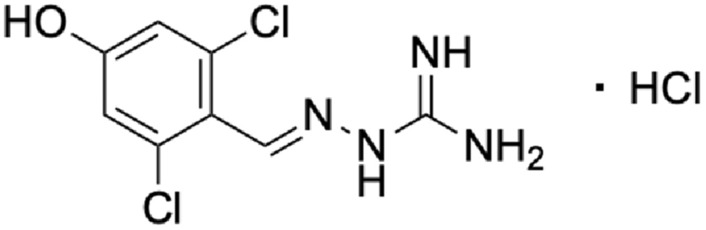
Chemical structure of 4-OH-Guanabenz. The structure was created using ChemDraw 20.1 software

4-Hydroxy guanabenz hydrochloride (4-OH-Guanabenz), full name: 2-[(2,6-Dichloro-4-hydroxyphenyl)methylene]hydrazinecarboximidamide Hydrochloride (Fig. 1) was synthesized in the Department of Medicinal Chemistry, Faculty of Pharmacy, Jagiellonian University Medical College, Kraków, Poland.

Heparin was delivered by Polfa Warszawa S.A. (Warsaw, Poland), while thiopental sodium and ketoprofen were from Sandoz GmbH (Austria), ketamine and xylasine from Biowet Puławy (Poland), and cefuroxime from Polfarma S.A. (Poland), brimonidine, oxymetazoline, and yohimbine from Sigma-Aldrich (Germany).

1-(3-Chlorophenyl)piperazine (mCPP) was synthesized in the Department of Organic Chemistry, Faculty of Pharmacy, Jagiellonian University Medical College, Kraków, Poland.

Chemicals, such as HPLC grade acetonitrile, methanol, and nebivolol, were supplied by Merck (Darmstadt, Germany). Formic acid was obtained from Fluka (Buchs, Switzerland). Potassium dihydrogen phosphate, disodium hydrogen phosphate, and sodium chloride were purchased from J. T. Baker (Phillipsburg, PA, USA). Purified water (18.2 MΩ) was delivered by a Milli-Q water system (Millipore, Billerica, MA, USA).

### Synthesis

Synthesis of 4-hydroxy guanabenz hydrochloride: chemical starting materials: 2,6-dichloro-4-hydroxybenzaldehyde (No: CDS004457-100MG), aminoguanidine hydrochloride (No: 396494) and solvents were purchased from Sigma-Aldrich (Poland), as reagent grade and used directly without further purification. NMR spectra were recorded on a JEOL spectrometer (JEOL Ltd., Tokyo, Japan) operating at 500 MHz (1H NMR) and 126 MHz (13C NMR). Chemical shifts, δ, are expressed in ppm and calculated taking reference METHANOL-d4. Ultra-performance liquid chromatography (UPLC)–mass spectrometry (MS) analysis was performed on the Waters ACQUITY UPLC (Waters Corporation, Milford, MA, USA) system coupled with a Waters tandem quadrupole (TQD) mass spectrometer (electrospray ionization (ESI) mode with TQD) using the C18 chromatographic column: particle size of 2.1 × 100 mm and 1.7 μm particle size. Gradient conditions: 95–0% eluent A for 10 min at a flow rate of 0.3 mL/min. Eluent A: 0.1% solution of formic acid in water (v/v); eluent B: 0.1% solution of formic acid in acetonitrile (v/v). Sample injection: 10 μL. Spectra were analyzed in the range of 200–700 nm. 4-OH-Guanabenz was synthesized according to the protocol: to a solution of 2,6-dichloro-4-hydroxybenzaldehyde (1 eq, 1.2 mmol, 0.226 g) in methanol (10 mL), aminoguanidine hydrochloride (1.25 eq, 1.5 mmol, 0.225 g) was added and the resulting reaction mixture was stirred at 95^°^ C for 3 h. After sufficient stirring, the reaction mixture was cooled to room temperature, and the final product was crystallized from methanol affording 2-[(2,6-dichloro-4-hydroxyphenyl)methylene]hydrazinecarboximidamide (pale salmon pink solid) with 95% yield (0.246 g). The compound was then transformed to hydrochloride salt by adding 2 M HCl solution in diethyl ether (10 mL) and stirring for 2 h at room temperature. After that time, the solid was filtered off affording final molecule as pale salmon pink solid: 2-[(2,6-Dichloro-4-hydroxyphenyl)methylene]hydrazinecarboximidamide hydrochloride: ^1^H NMR (METHANOL-d4, 500 MHz) δ 8.35 (s, 1H), 6.90 (s, 2H), NH protons not detected. ^13^C NMR (METHANOL-d4, 126 MHz) δ 159.6, 155.8, 143.6, 135.6, 119.9, 116.1. Chemical Formula: C_8_H_9_C_l3_N_5_O; detected: MS (ESI +) *m*/*z*: 246.96 [M + H^+^]. Purity: 100% (UPLC-UV-MS).

Supplementary data: spectral data of 4-hydroxy guanabenz hydrochloride.

### Experiment methods

#### In silico preliminary docking studies

Structures of Guanabenz and 4-OH-Guanabenz in SMILES format were used to predict possible biological targets using Swiss Target Prediction [23]. Possible similarity to active ligands in ChEMBL database was determined using a modified open KNIME workflow [24]. For this purpose, structures were encoded in Morgan/EFCP2 fingerprints from the SMILES format provided. Ligand’s possible protonation states at pH = 7.4 were determined using Marvin [25] and prepared using LigPrep [26].

For molecular docking purposes, the crystal structure of serotonin 5-HT_2C_ receptor (PDB ID: 6BQG) and an AlphaFold2 model of trace amine-associated receptor 1 (TAAR_1_) (AF-Q96RJ0) were used. Proteins were pre-processed using Protein Preparation Workflow [27, 28]. The receptor grid (box of 16 Å) was determined on the basis of the bound ligand (5-HT_2C_), on the basis of PrankWeb predictions [28]. Docking to a rigid form of the protein was performed using the Glide module [29], with default settings. Visualizations were prepared using Schrödinger Maestro [26].

#### Adrenoceptor-binding assay

##### Preparation of solutions of test and reference compounds

10 mM stock solutions of test compounds were prepared in DMSO. Serial dilutions of compounds were prepared in 96-well microplate in assay buffers using an automated pipetting system epMotion 5070 (Eppendorf, Poland). Each compound was tested in 6 concentrations of 1.0E-05 to 1.0E-10 M (final concentration).

##### Receptor-binding assay

Affinity towards α_1_- and α_2_-adrenoceptors was evaluated using the competitive radioligand-binding assay. Experiments were conducted in the rat cerebral cortex. Radioligand [3H]prazosin (84,2 Ci/mmol, α_1_-adrenoceptor, PerkinElmer, Poland, NET823001MC) and [3H]clonidine (70.5 Ci/mmol, α_2_-adrenoceptor, PerkinElmer, Poland, NET613250UC) were used. The membrane preparation and the assay procedure were carried out accordingly as previously published [30]. Radioactivity was counted on the MicroBeta2 scintillation counter (PerkinElmer, Poland). Data were fitted to a one-site curve-fitting equation with Prism 8 (GraphPad Software, USA), and K_i_ values were estimated from the Cheng–Prusoff equation.

#### The intrinsic activity at α_2A_, α_2B_-adrenoceptors and TAAR_1_

The intrinsic activity at α_2A_-adrenoceptor was assessed by the Tango assay technology that uses a mammalian-optimized beta-lactamase (bla) reporter gene combined with an FRET-enabled substrate to provide reliable and sensitive detection in cells. Cells are loaded with an engineered fluorescent substrate containing two fluorophores, coumarin, and fluorescein. In the absence of bla expression, the substrate molecule remains intact. In this state, excitation of the coumarin results in a transfer of fluorescence resonance energy to the fluorescein moiety and emission of green fluorescent light. However, in the presence of bla expression, the substrate is cleaved, separating the fluorophores and disrupting energy transfer. Excitation of the coumarin in the presence of bla enzyme activity results in a blue fluorescence signal.

Intrinsic activity assay was performed according to the manufacturer of the assay kit (Invitrogen, Life Technologies). Cells were harvested and resuspended in an assay medium at a density of 312,500 cells/ml. 32 μL per well of cell suspension was added to the test compound wells, the unstimulated control wells, and the stimulated control wells, and incubated for 16–24 h. To perform an agonist assay, 8 μL of 5 × of agonist in the assay medium was added to the cells. To perform an antagonist assay, 4 μL of 10 X antagonist in assay medium was added to the cells, and after 30 min, 4 μL of 10 X standard agonist in EC_80_ (Brimonidine 10^–7^ M) in assay medium was added to the cells. Then, the agonist and antagonist plates were incubated in a humidified incubator at 37 °C/5% CO_2_ for 5 h. After the incubation, 8 μL of LiveBLAzer™-FRET B/G substrate mixture (CCF4-AM) was loaded into cells in the absence of direct strong lighting, covered, and incubated at room temperature for 2 h.

The intrinsic activity at α_2B_-adrenoceptor was evaluated by luminescence detection of calcium mobilization using the recombinant expressed jellyfish photoprotein, aequorin.

Measurements were performed with the α_2B_-adrenergic AequoScreen cell line (PekinElmer cat. no. ES-031-AF). Cell density, in 96-well format measurements, was 5000 cells per well. Cell harvesting, coelenterazine h (Invitrogen, cat. no. C 6780) loading, and preparation were performed according to the instructions presented in the AequoScreen Starter Kit Manual (PerkinElmer, Poland). Compound concentration series (50 μL/well) were diluted in 0.1% BSA (Intergen, cat. no. 3440–75) containing assay buffer (D-MEM/F-12, Invitrogen cat. no. 11039) and prepared in white ½ Area Plate—96-well microplates (PerkinElmer, cat. no. 6005560). The cell suspension was dispersed on the ligands using POLARstar optima reader injectors (BMG Labtech, Germany).

Oxymetazoline (Sigma, Cat. no. O2378) was used as an agonist for the α_2B_-adrenoceptors. The concentration and dilution series having four replicates were prepared as instructed in the AequoScreen Starter Kit Manual. Cells were injected into wells with standard agonist or tested compounds using the POLARstar optima reader (BMG Labtech, Germany), and emitted light was recorded for 20 s. For the antagonist assay, cells were injected (50 μL) into the assay plate with antagonists (50 μL). Agonist (Oxymetazoline) was injected at a single concentration (50 μL, final concentration EC_80_) on the preincubated (15–20 min) of cells + antagonist and the emitted light was recorded for 20 s.

The intrinsic activity study at cells with overexpression of TAAR_1_ (Eurofins, cat. No. 95-0173C2) was carried out at Eurofins Discoveries (Fremont, USA) using the Hit Hunter cAMP method. This assay monitors the activation of a GPCR via Gi and Gs secondary messenger signaling in a homogenous, non-imaging assay format using a technology developed by DiscoverX called Enzyme Fragment Complementation (EFC) with β-galactosidase (β-Gal) as the functional reporter. The enzyme is split into two complementary portions: EA for Enzyme Acceptor and ED for Enzyme Donor. ED is fused to cAMP and in the assay competes with cAMP generated by cells for binding to a cAMP-specific antibody. Active β-Gal is formed by complementation of exogenous EA to any unbound EDcAMP. The active enzyme can then convert a chemiluminescent substrate, generating an output signal detectable on a standard microplate reader.

cAMP Hunter cell lines were expanded from freezer stocks according to standard procedures. Cells were seeded in a total volume of 20 µL into white-walled, 384-well microplates and incubated at 37 ℃ for the appropriate time prior to testing. For agonist determination, cells were incubated with a sample to induce a response. Media was aspirated from cells and replaced with 15 µL 2:1 HBSS/10 mM Hepes:cAMP XS + Ab reagent. Intermediate dilution of sample stocks was performed to generate 4X samples in assay buffer. 5 µL of 4 × sample was added to cells and incubated at 37 ℃ or room temperature for 30 or 60 min. Vehicle concentration was 1%. After appropriate compound incubation, the assay signal was generated through incubation with 20 µL cAMP XS + ED/CL lysis cocktail for 1 h followed by incubation with 20 µL cAMP XS + EA reagent for 3 h at room temperature. Microplates were read following signal generation with a PerkinElmer Envision instrument for chemiluminescent signal detection. Intrinsic agonist activity was determined at a maximum concentration of 10^–4^ M, computer simulation (GraphPad Prism, USA) was used to calculate the EC_50_ value, and for concentrations of 10^–2^ and 10^–1^ M, the response was assumed to be 90–100% of the maximum tyramine response.

#### Obesity induced with a high-fat diet and the influence of the tested compound on body weight

Male Wistar rats were housed in pairs and fed a high-fat diet consisting of a 40% fat blend (Labofeed B with 40% lard, Morawski, Manufacturer Feed, Poland) for 14 weeks, with water available ad libitum [7, 31, 32]. Control rats were fed a standard diet (Labofeed B, Morawski Manufacturer Feed, Poland). After 10 weeks, rats with obesity-induced via their diet were randomly divided into three equal groups that had the same mean body weight and were treated *ip* with test compounds at doses of 2 or 5 mg/kg b.w./day (high-fat diet + 4OHGuanabenz) or control group: vehicle–water 0.3 ml/kg (high-fat diet + vehicle = obesity control group) once a day in the morning between 9:00 AM and 10:00 AM for 25 days. Control rats were maintained on a standard diet, with *ip* administration of vehicle – water (standard diet + vehicle = control group). We chose the doses of the tested compound—2 or 5 mg/kg b.w. based on our previous experiments with guanabenz [18].

High-fat feeding composition (932 g of dry mass): protein—193 g, fat (lard)—408 g, fiber—28.1 g, crude ash—43.6 g, calcium—9.43 g, phosphorus—5.99 g, sodium—1.76 g, sugar—76 g, magnesium—1.72 g, potassium—7.62 g, manganese—48.7 mg, iodine—0.216 mg, copper—10.8 mg, iron—125 mg, zinc—61.3 mg, cobalt—0.253 mg, selenium—0.304 mg, vitamin A—15000 units, vitamin D3—1000 units, vitamin E—95.3 mg, vitamin K3—3.0 mg, vitamin B1—8.06 mg, vitamin B2—6.47 mg, vitamin B12—0.051 mg, folic acid—2.05 mg, nicotinic acid—73.8 mg, pantothenic acid—19.4 mg, and choline—1578 mg.

The high-fat diet contained 550 kcal and the standard diet 280 kcal per 100 g.

#### Effect on food intake

Food intake was measured three times a week, immediately before drug administration. In this target, food was provided to the animals in a certain amount (100 g/cage). The weight of food was measured using dedicated scale (WPT 1C, RADWAG Wagi Elektroniczne, Poland). Next, the total amount of food that the animals had not eaten during that period of time was collected from each cage and weighed, and then, the data were recorded. The next portion of food was subsequently weighed before serving, and so on.

#### Effect on spontaneous activity (monitoring the movement of the rat in the home cage under standard breeding conditions)

Rat spontaneous activity was monitored for 24 h on the 1st and 24th (after multiple administrations of tested compound or vehicle) day of treatment with a radio-frequency identification system (RFID system)—TraffiCage (TSE-Systems, Germany)—transponder-based activity monitoring under group housing [7, 33]. The animals had subcutaneously implanted transponder identification with RFID code, which allowed the presence and time spent in different areas of the home cage to be recorded. The data were collected with a dedicated platform, placed under the home cage, containing five RFID antennas (six cage areas) reading telemetrically the presence of RFID chips and stored for analysis with a dedicated computer program. The animals during the monitoring of spontaneous activity were in home cages and always had free access to food and water.

#### Collecting plasma and adipose tissue

On the 26th day of the experiment, 20 min after ip administration of heparin (5000 IU/rat) and thiopental (70 mg/kg b.w.), plasma was collected from the left carotid artery and then centrifuged at 600 × g (15 min, 4 °C) to obtain the plasma.

#### Effect on glucose, triglyceride, and total cholesterol levels in the plasma of obese rats

To determine the glucose, triglyceride, or cholesterol level in plasma, standard enzyme and spectrophotometric tests (Biomaxima S.A. Lublin, Poland) were used. The substrate was decomposed with enzymes appropriate for the relevant product, which was converted to a colored compound. The colouration was proportional to the concentration. The absorbance was measured at a wavelength of 500 nm.

#### Effect on body weight of non-obese rats fed only with a standard diet

Male Wistar rats (190–220 g) were housed in pairs. The control group (standard diet + vehicle) received vehicle (water, *ip*), while the test group (standard diet + 4-OH-Guanabenz) was injected (*ip*) with 4-OH-Guanabenz at a dose of 5 mg/kg b.w. dissolved in water. Body weights were measured daily immediately prior to drug administration.

#### Effect on core body temperature of obese rats

Animals with induced obesity (two more groups) were subcutaneously implanted with a DST micro-HRT heart rate logger (Star-Oddi, Island), which simultaneously measured long-term core temperature. Under general anesthesia (thiopental, 70 mg/kg, *ip*), the loggers were inserted under the skin in the groin area and sutured with a surgical thread. Forty-eight hours later, the baseline temperature was measured, and at 9:30 AM, 4-OH-Guanabenz was administered *ip* at a dose of 5 mg/kg b.w. The first measurement was recorded 30 min after the administration of the test compound, and then, the temperature was recorded every hour. After 24 h, the loggers were removed, the temperature data were read, and Mercury software (Star-Oddi Mercury Data Logger PC Software, Iceland) was used to collect and analyze the information obtained [7]. Using this technology, measurements are made without the direct intervention of the researcher; therefore, there is no disturbance in the readings evoked by stress in the animals.

#### Effect on blood pressure of non-obese, normotension rats

Eighteen normotensive rats were anaesthetized with thiopental (70 mg/kg) by *ip* injection. The left carotid artery was cannulated with tubing filled with heparin solution in saline to facilitate pressure measurements using Apparatus PowerLab 4/35 (ADInstruments, Australia). Blood pressure was measured: before *ip* administration of 4-OH-Guanabenz at the doses of 2 or 5 or 10 mg/kg b.w. or water – time 0 min (control pressure) and continuously during the next 60 min.

#### Effect on blood pressure during a 24-h study in non-obese, normotension rats residing in natural housing conditions: telemetric method

The blood pressure of rats treated with the tested compound was measured for a 24 h period after *ip* treatment (5 mg/kg b.w.) with a special telemetric system Stellar (TSE-Systems, Germany) [34, 35].

Surgery: The operation was carried out over 30 min under sterile conditions. Rats were anaesthetized with ketamine and xylasine (intramuscular injection: 100 mg/kg and 10 mg/kg). Before surgery and for 7 days after, the animals were also treated with cefuroxime (20 mg/kg/day) via intramuscular injection and ketoprofen (5 mg/kg/day) via* ip* injection. The blood flow in the abdominal aorta was temporarily blocked and the tip of a transmitter was inserted to measure pressure. The transmitter was sutured to the peritoneal cavity.

The rats were individually caged for 2 weeks to heal after surgical cut. The animals were then placed in pairs in cages to reduce their isolation stress. Blood pressure was measured: before *ip* administration of the compounds – time 0 min and 24 h thereafter.

#### Pharmacokinetic analysis

The pharmacokinetic parameters were calculated using a non-compartmental approach from the average concentration values, using Phoenix WinNonlin software (Certara, Princeton, NJ 08540 USA). The first-order elimination rate constant (λ_z_) was calculated by linear regression of time versus log concentration. Next, the area under the mean plasma and the concentration in the brain versus the time curve (AUC_0→t_) were estimated using the log-linear trapezoidal rule (Eq. 1), where Cn is the concentration of the last sampling of each compound

1 $${\text{AUC}}_{0 \to t} = \mathop \sum \limits_{i = 1}^{n} \left( {(C_{i} + C_{i + 1} )/2} \right) \cdot \left( {t_{i + 1} - t_{i} } \right) + C_{n} /\lambda_{z} .$$

The area under the first-moment curve (AUMC_0→t_) was estimated by calculating of the total area under the first-moment curve using Eq. 2, where t_n_ is the time of last sampling

2 $${\text{AUMC}}_{0 \to t} = \mathop \sum \limits_{i = 1}^{n} \left( {(t_{i} \cdot C_{i} + t_{i + 1} \cdot C_{i + 1} )/2} \right) \cdot \left( {t_{i + 1} - t_{i} } \right) + (t_{n} \cdot C_{n} )/\lambda_{z} + C_{n} /\lambda_{z}^{2} .$$

Mean residence time (MRT, Eq. 3) was calculated as

3 $${\text{MRT}}\,\, = \,\,\frac{{{\text{AUMC}}_{{{0} \to t}} }}{{{\text{AUC}}_{{{0} \to t}} }}.$$

Total clearance (Cl_T_, Eq. 4) was calculated as

4 $$C1_{T} \, = \,\frac{{F \cdot D_{i.p.} }}{{{\text{AUC}}_{0 \to t} }}.$$

The volume of distribution (V_d_, Eq. 5) was calculated as

5 $$V_{d} \, = \,\frac{F \cdot D}{{\lambda_{{\text{z}}} {\text{AUC}}_{0 \to t} }},$$

where *D*_*ip*_ is an *ip* dose of Guanabenz and 4-OH-Guanabenz.

##### Analytical method

The quantification of compounds in plasma and brain samples was performed using the Sciex API 3200 triple quadrupole mass spectrometer (Concord, Ontario, Canada) equipped with an electrospray (ESI) ionization interface. This instrument was coupled to an Elite LaChrom (Hitachi, USA) HPLC system. Data acquisition and processing were performed using Sciex Analyst 1.5.2 data collection and integration software. After preparation, the samples were injected (20 µL) onto XBridge C18 (2.1 mm × 30 mm, 3.5 μm, Waters, Milford, Massachusetts, USA) analytical column. The mobile phase consisted of 0.1% formic acid in acetonitrile (A) and 0.1% formic acid in water (B) was delivered in a gradient elution starting with 90% of eluent B, increasing for 2 min to 90% of eluent A, maintained for 3 min, and then returned for 5 min to 90% of eluent B, and maintained 90% of eluent B during 5 min at a flow rate of 300 µL/min. The total analysis time was 10 min. The electrospray ionization process was performed in positive ionization mode and data acquisition was carried out in selected reaction monitoring mode (SRM) for guanabenz and 4-OH-Guanabenz and its internal standard (carvedilol). The ion spray source settings were as follows: spray voltage: 5.5 kV, heater temperature: 400 °C, curtain gas: 10 psi, source gas 1: 20 psi, and source gas 2: 20 psi. The measured ions were: *m*/*z* 232 (Q1) and *m*/*z* 215.1(Q3) for guanabenz; *m*/*z* 247 (Q1) and *m*/*z* 187.9 (Q3) for 4-OH-Guanabenz, and *m*/*z* 407.2 (Q1) and *m*/*z* 100.1 (Q3) for carvedilol (IS), Table 1.

**Table 1:** Selected mass spectrometry parameters optimized for guanabenz, 4-OH-Guanabenz and carvedilol

Analyte	MRM (m/z)	CE [V]	DP [V]	EP [V]	CXP [V]
Guanabenz	232 → 215.1	39	34	7	16
4-OH-Guanabenz	247 → 187.9	40	45	8	17
IS (Carvedilol)	407.2 → 100.1	33	50	6	15

*CE* collision energy, *DP* declustering potential, *EP* entrance potential, *CXP* collision cell exit potential

##### Preparation of standard solutions

One mg of guanabenz and 1 mg of 4-OH-Guanabenz were accurately weighted and transferred to the 1 mL volumetric flask, dissolving in methanol as the solvent. Further dilutions were performed using the same solvent to prepare working standard solutions of the analyte at the following concentrations: 0.025, 0.05, 0.1, 0.25, 0.5, 1.0, 2.5, 5.0, 10, 25, and 50 µg/mL for calibration curve samples (CC) and 0.025, 0.075, 2.2, and 4.5 µg/mL for quality control samples (QC). To prepare samples for calibration curve or quality control samples, 45 µL of the matrix (plasma or brain homogenate) was added with 5 µL of internal standard solution (1 µg/mL) obtaining the final concentration of 100 ng/mL and 5 µL of standard working solutions at the needed CC or QC concentration levels. After the addition of the standard solution, the samples were mixed and purified.

##### Sample preparation

A sample volume of 50 µL of plasma or brain homogenate was transferred to the clean Eppendorf tube and spiked with 5 µL of IS solution (1 µg/mL) obtaining the final concentration of 100 ng/mL. After 5 min of mixing (1500 rpm) proteins were precipitated using 200 µL ACN. After 10 min of sample shaking (1500 rpm), the incubation step was performed (10 min, 4 °C). The samples were then centrifuged (10,000 rpm, 10 min, 4 °C) and the supernatant was transferred to chromatographic vials for LC/MS/MS analysis.

Brain homogenate was prepared to maintain tissue: phosphate-buffered saline (PBS) at a ratio of 1:5. Homogenisation was carried out using an IKA® T10 Basic ULTRA-TURRAX disperser (IKA Werke GmbH & Co. KG, Staufen, Germany). After homogenisation 50 µL of sample was collected for further preparation. All samples were stored on ice during the preparation process.

### Statistical analysis

Statistical analysis was performed using GraphPad Prism 6 software (GraphPad Software, USA). The normality of the data sets was determined using the Shapiro–Wilk test. Comparisons between the experimental and control groups were performed by one-way (treatment—an independent variable: sum of body weight change, and biochemical parameters) or two-way ANOVA (treatment—an independent variable and time as a repeated measure: body weight, food intake, core temperature, blood pressure, and spontaneous activity), followed by Tukey or Bonferroni post hoc. Differences were considered significant at: *p* ≤ 0.05.

## Results

### In silico biological target predictions

Using the online Swiss Target Prediction tool [23], possible biological targets for 4-OH-Guanabenz were evaluated and are gathered in Table 2.

**Table 2:** Biological targets predicted for 4-OH-guanabenz by Swiss target prediction

Target	Probability
Alpha-2a adrenergic receptor	0.0619466223505
Adrenergic receptor alpha-2	0.0619466223505
Alpha-2b adrenergic receptor	0.0619466223505
Alpha-1d adrenergic receptor	0.0619466223505
Nischarin	0.0619466223505
Alpha-1a adrenergic receptor (by homology)	0.0619466223505
Trace amine-associated receptor 1	0.0619466223505
Monoamine oxidase A	0.0524239602665
Serotonin 2a (5-HT2a) receptor	0.0428942118511
Serotonin 2b (5-HT2b) receptor	0.0428942118511
Serotonin 2c (5-HT2c) receptor	0.0428942118511

**Fig. 2:**
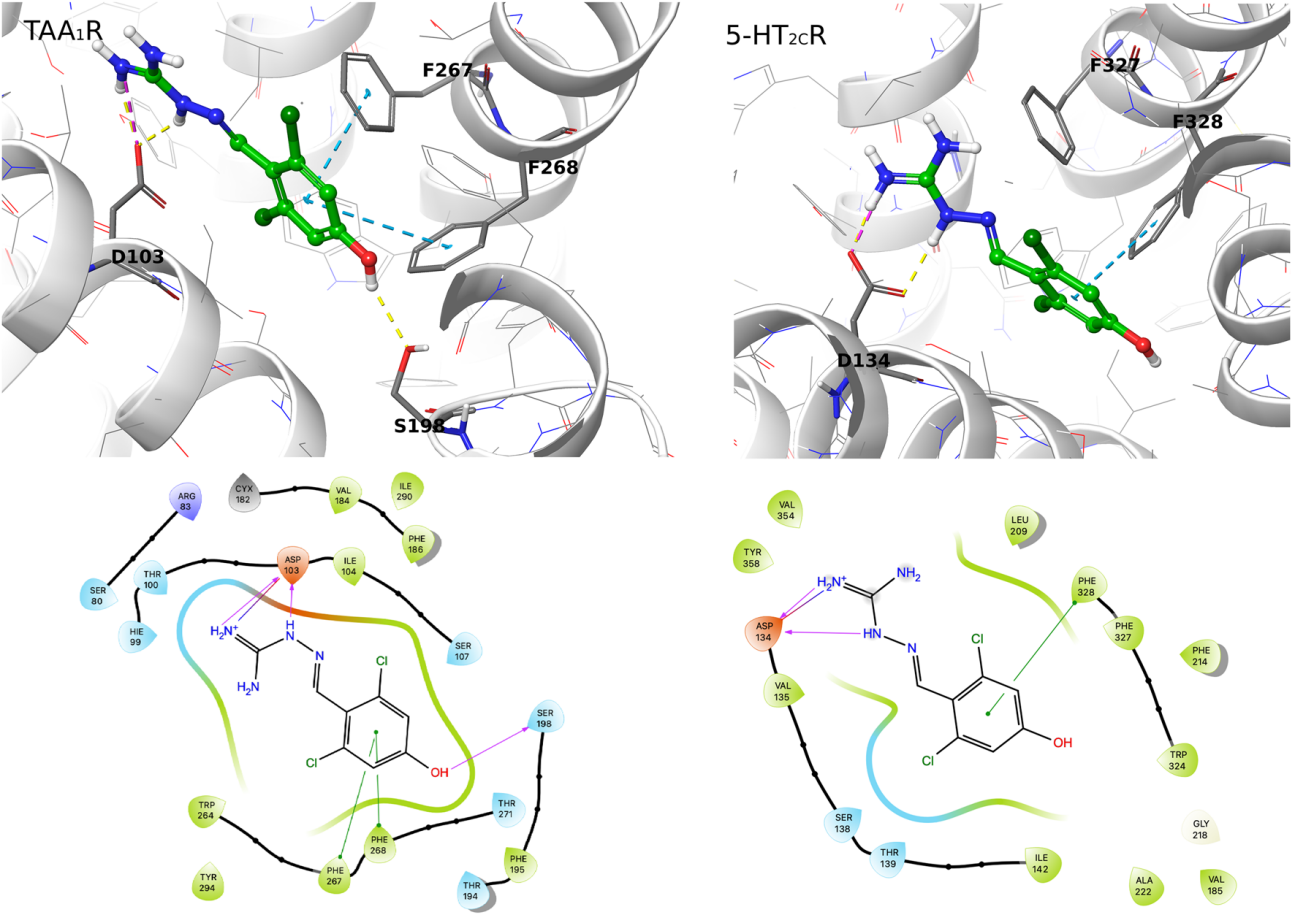
Putative binding poses of 4-OH-Guanabenz in orthosteric binging pocket of TAAR_1_ (upper left panel) and serotonin 5-HT_2C_ receptor (upper right panel) with corresponding ligand interaction diagrams (lower panel)

To confirm the possible interaction with trace amine-associated receptor 1 (TAAR_1_), a similarity search to known TAAR_1_ ligands was performed in the ChEMBL database and resulted in one hit: ChEMBL420 (Guanabenz) with a Tannimoto coefficient of 0.68. Preliminary docking studies to the TAAR_1_ model confirmed the target predictions. The putative, calculated binding mode of 4-OH-Guanabenz appeared in the perpendicular plane to the cell membrane, interacting through the protonated guanidine fragment with the key D103 forming H-bonds and/or a salt bridge. The hydroxyl group at position 4 was facing TM6 and forming a H-bond with S198, while the benzene ring was stabilized by stacking interactions with F267 and F268, followed by hydrophobic interactions with I104, I290, V184, F195, and W264 (Fig. 2, upper left panel). The results were in agreement with, for example, the study of Francesconi et al. and proved the possible affinity of the tested compound to TAAR_1_ [36].

A similar binding mode was found when docked to the serotonin 5-HT_2C_ receptor—the guanidine nitrogens formed H-bond and/or salt bridge with D134, while the benzene ring was stabilized by stacking interactions with F328, followed by hydrophobic interactions with V135, I142, F214, and W324 (with distances of 3.88, 4.40, 4.04, and 4.12 Å, respectively; Fig. 2, upper right panel). A slightly different conformation with rotated guanidine fragment interacting additionally through cation-π with F327 was also found.

### Affinity towards α-adrenoceptors

4-OH-Guanabenz possesses a high affinity toward α_2_-adrenoreceptor. Additionally, the tested compound had an almost 200-fold lower affinity for the α_1_-adrenoceptor subtype (Table 3).

**Table 3:** Affinity towards α-adrenoceptors

	α_1_-Adrenoceptor	α_2_-Adrenoceptor
	[3H] prazosin	[3H] clonidine
	Ki [nM]	Ki [nM]
4-OH-Guanabenz	3856.0 ± 1007.0	19.4 ± 0.7
Guanabenz	253.3 ± 2.8	3.2 ± 0.4
Phentolamine	11.3 ± 1.7	NT
Phenylephrine	NT	146.0 ± 20.0

Mean ± SEM, *n* = 3

*NT* not tested

### Intrinsic activity at α_2A_-, α_2B_-adrenoceptors and TAAR_1_

**Fig. 3:**
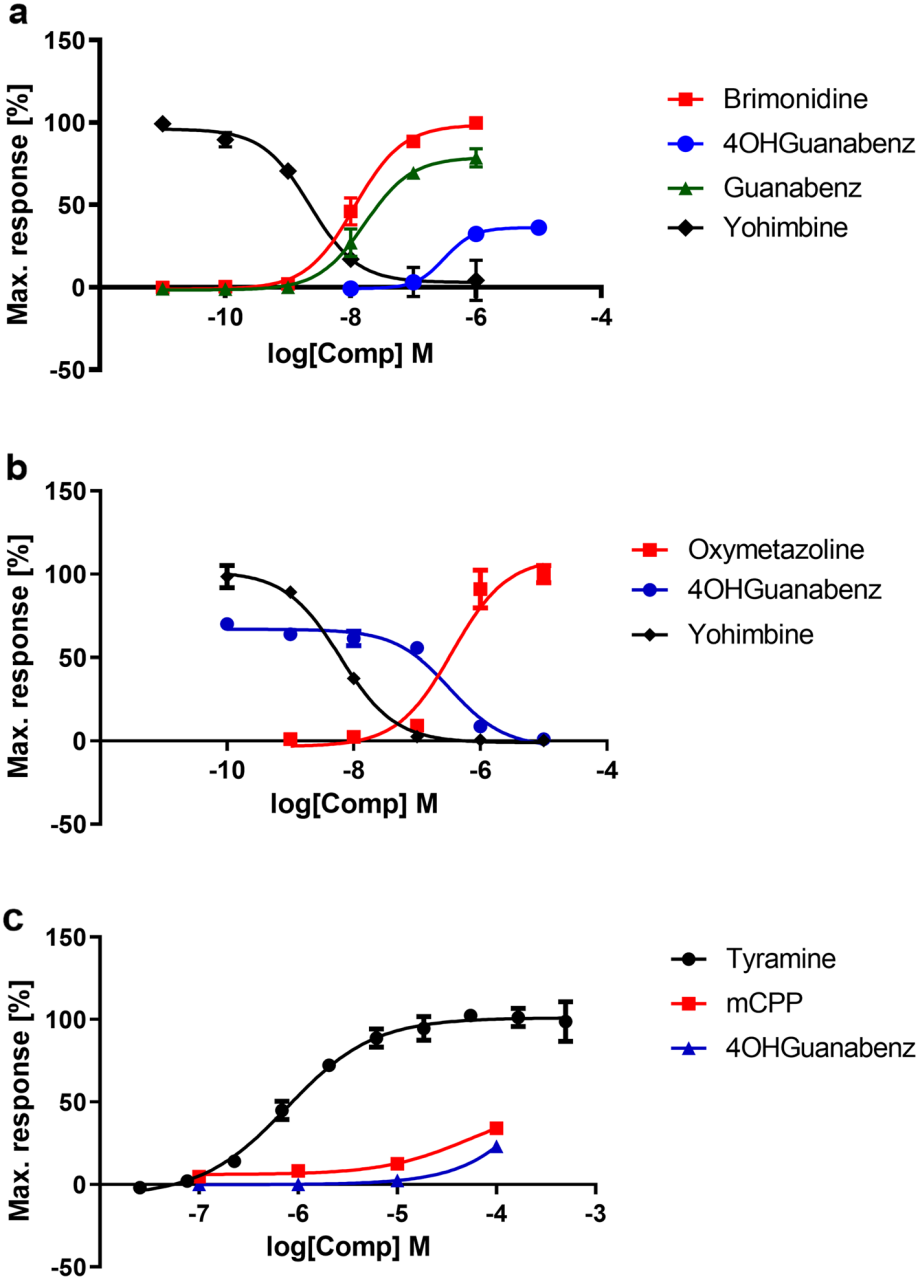
The intrinsic activity at α_2A_-adrenoceptor (**a**), α_2B_-adrenoceptor (**b**), and TAAR_1_ (**c**) of the tested compounds presented as concentration-dependence curves. (**a**) Obtained values (%) are expressed as percent of the action of full agonist brimonidine at the dose of EC_80_ (100%), (**b**) obtained values (%) are expressed as percent of the action of full agonist oxymetazoline at the dose of EC_80_ (100%), and (**c**) obtained values (%) are expressed as percent of the action of full agonist tyramine at the dose of EC_80_ (100%)

4-OH-Guanabenz showed the activity of a partial agonist of α_2A_-adrenoceptor reaching 30% of the activity of a full agonist brimonidine. The calculated EC_50_ value for 4-OH-Guanabenz was 316.3 nM and was approximately 27 times higher than the EC_50_ of brimonidine, for which the EC_50_ value was estimated at 11.5 nM. Guanabenz was found to be a full agonist at the α_2A_-adrenoceptor and the calculated EC_50_ was 16.32 nM. Its agonist activity was similar to that of the reference compound brimonidine. Yohimbine was used as a reference compound with antagonistic properties, for which the IC_50_ value was 2.26 nM (Fig. 3a).

4-OH-Guanabenz showed antagonist activity at α_2B_-adrenoceptor. The calculated EC_50_ value for 4-OH-Guanabenz was 330.2 nM and was approximately 54 times higher than the EC_50_ of yohimbine for which the EC_50_ value was estimated at 6.13 nM. Oxymetazoline was used as a reference compound with agonistic properties, for which the IC_50_ value was 359.6 nM (Fig. 3b).

4-OH-Guanabenz and mCPP (known agonist of TAAR_1_ [37]) showed weak agonistic activity at TAAR_1_. At the highest concentration tested, i.e., 10^–4^ M, both tested compounds achieved 23% and 34% of the activity of the full agonist tyramine, respectively. The EC_50_ values for mCPP and 4-OH-Guanabenz calculated after computer simulation were 236.6 µM and 330.6 µM, respectively (Fig. 3c).

### Influence on body weight and caloric intake and spontaneous activity of obese rats

**Fig. 4:**
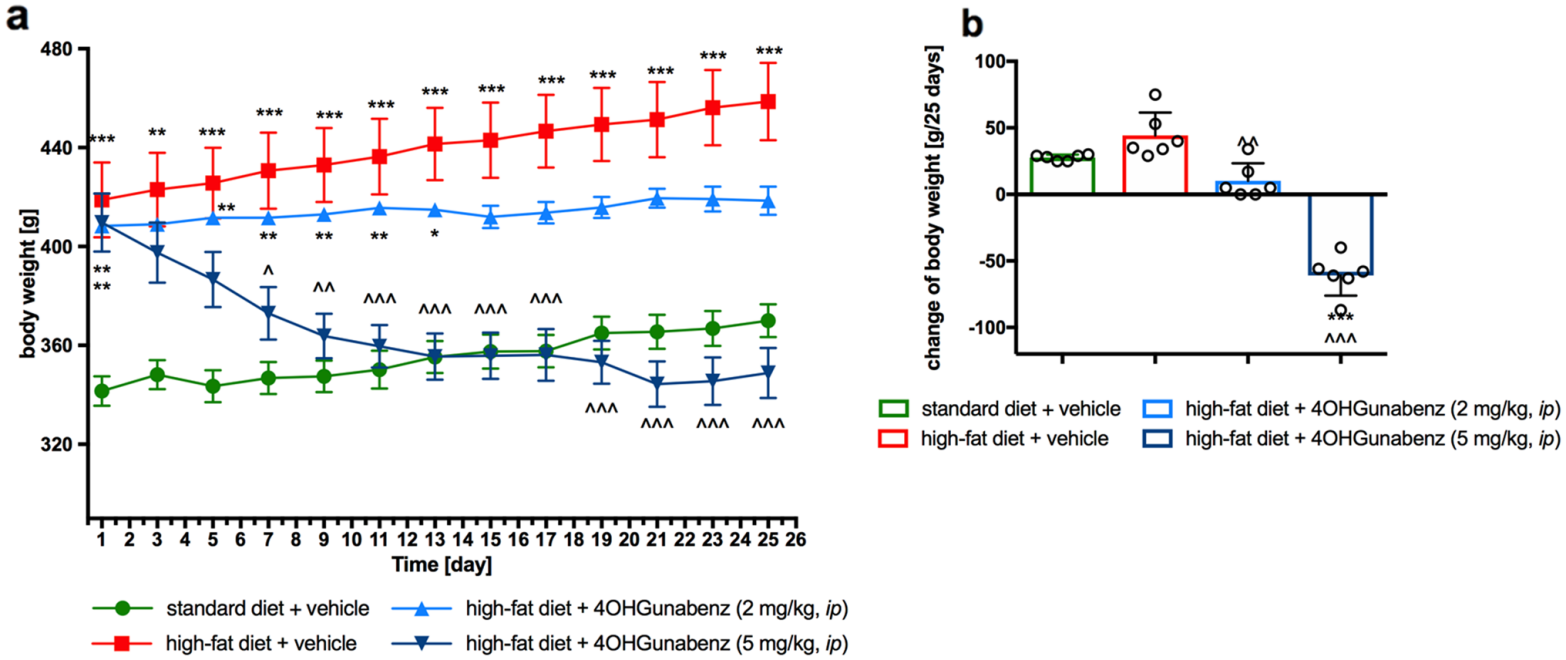
The effect of 4-OH-Guanabenz on body weight in male Wistar rats fed with a high-fat diet. Daily changes in body weight in control (standard diet) and diet-induced obese rats, and diet-induced obese rats treated for 25 days with the tested compound (**a**), the sum of the change in body weight (**b**). Mean ± SEM, *n* = 6 (**a**) or mean ± SD (**b**). Multiple comparisons were performed by two-way ANOVA with repeated measure, Tukey post hoc test (**a**), or by one-way ANOVA, Tukey post hoc test (**b**). *p < 0.05, **p < 0.01, ***p < 0.001 significant *vs*. control rats fed standard diet; ^*p* < 0.05, ^^*p* < 0.01, ^^^*p* < 0.001 significant *vs*. control rats fed a high-fat diet (diet-induced obesity control group)

4-OH-Guanabenz administered *ip* at a dose of 5 mg/kg b.w. to rats fed a high-fat diet caused a significant decrease in body weight compared to the control group fed the same feed (obese rats) (Fig. 4). The weight loss in this group treated with the tested compound was approximately 14.8% compared to the baseline weight before treatment. However, the compound that was administered at a dose of 2 mg/kg b.w. did not cause weight loss, but significantly reduced weight gain compared to the weight gain determined in the control group fed the same feed (*F*_3,20_ = 73.04, *p* < 0.0001, two-way ANOVA) (Fig. 4).

**Fig. 5:**
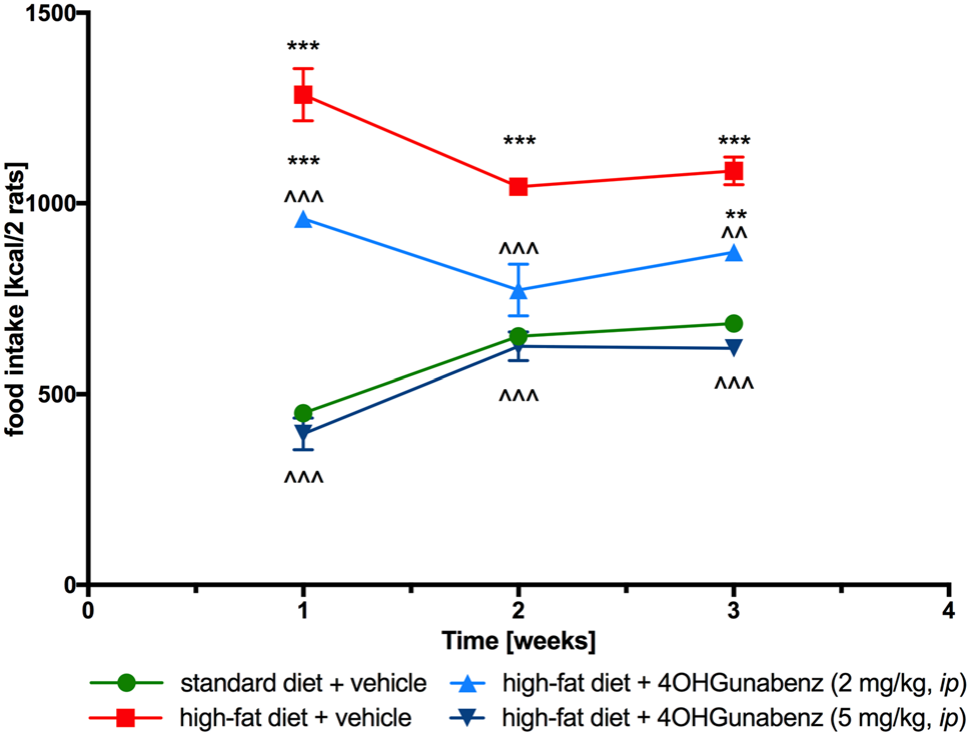
The effect of 4-OH-Guanabenz on food intake in male Wistar rats fed with a high-fat diet in weeks 1–3 and throughout the experiment. Mean ± SEM, *n* = 3, data for two animals housed together. Multiple comparisons were performed by two-way ANOVA for repeated measures, Tukey post hoc test. **p < 0.01, ***p < 0.001 significant *vs*. control rats fed standard diet; ^^^*p* < 0.001 significant *vs*. control rats fed a high-fat diet (diet-induced obesity control group)

4-OH-Guanabenz administered *ip* at both doses reduced the number of calories consumed by rats in the test group compared to the obesity control group (Fig. 5). The effect when administering a dose of 2 mg/kg b.w. was less intense. It was only in the second week of treatment where the amount of calories consumed by the animals did not differ from the amount of calories consumed in the control group fed with standard feed.

**Fig. 6:**
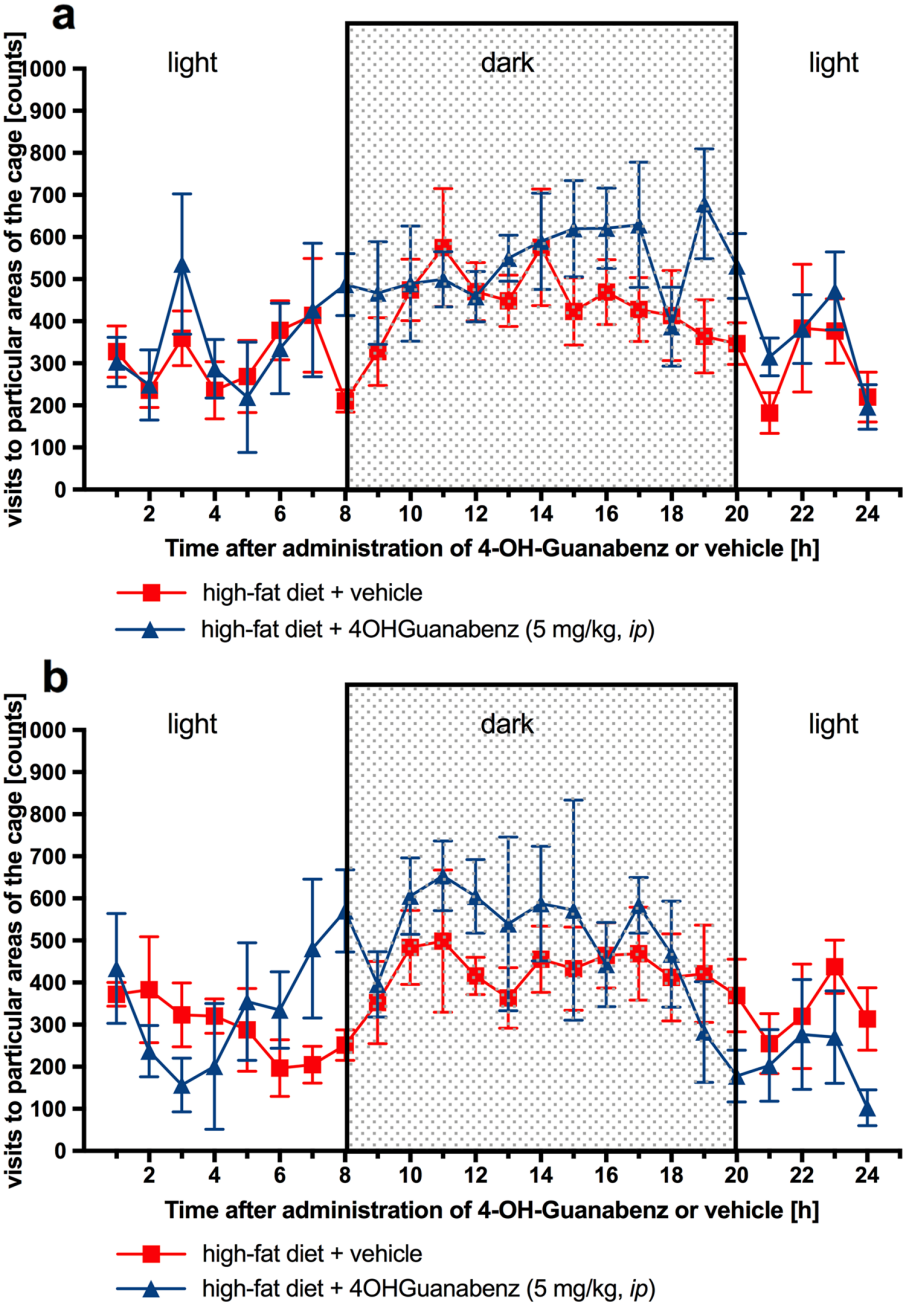
The effect of 4-OH-Guanabenz on spontaneous activity in male Wistar rats fed with high-fat diet *(monitoring the movement of the rat in the home cage under standard breeding conditions)*. Activity during 24 h after the first administration of 4-OH-Guanabenz (**a**) or activity during 24 h after the 24th administration of 4-OH-Guanabenz (**b**). Mean ± SEM, *n* = 4–6. Comparisons were performed by two-way ANOVA with repeated measure

The monitoring of spontaneous activity in obese animals showed no effect after the first and 24th administrations of 4-OH-Guanabenz at a dose of 5 mg/kg b.w. (Fig. 6).

### Influence on plasma glucose, triglyceride, or total cholesterol levels of obese rats

**Fig. 7:**
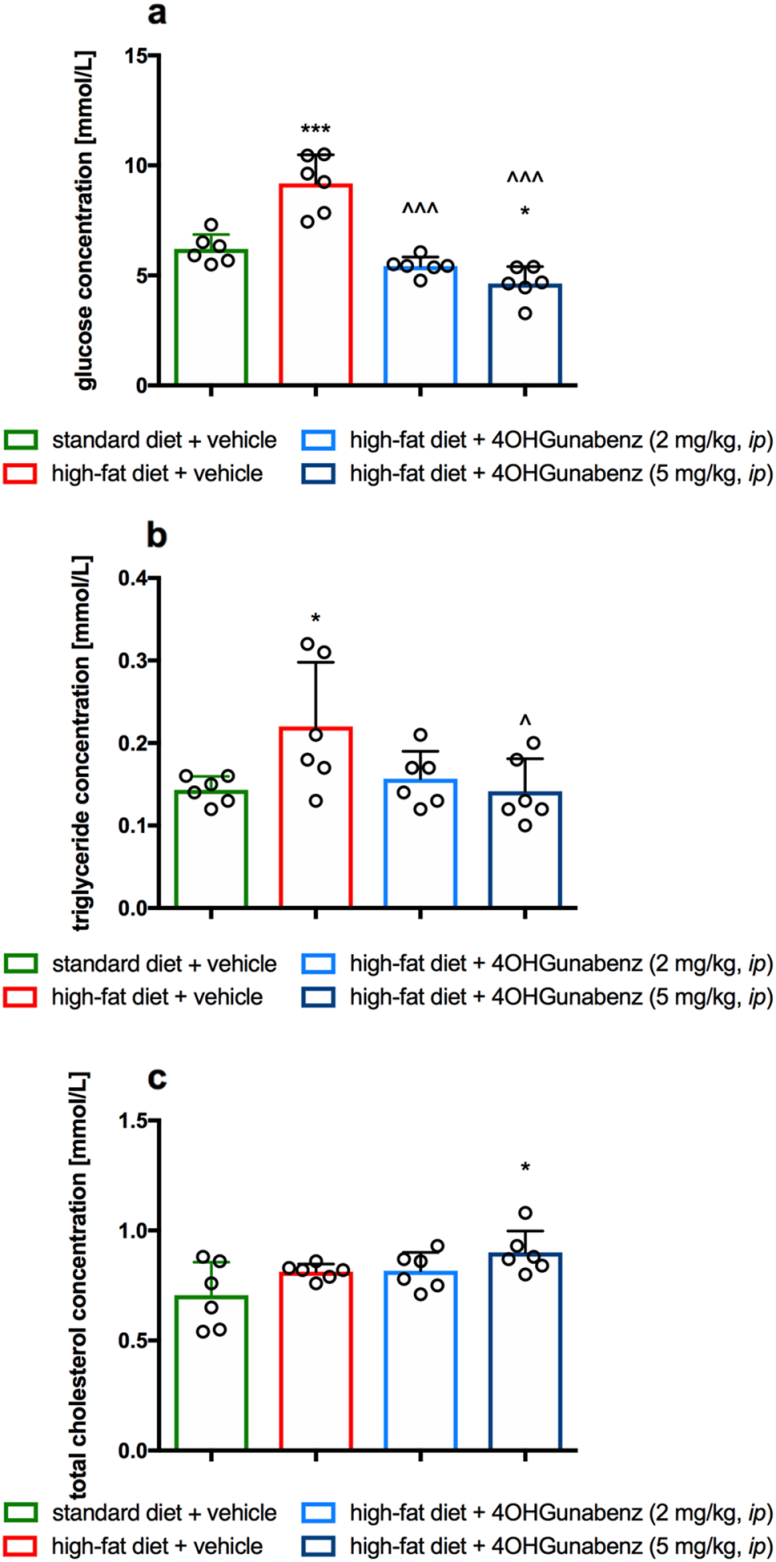
The effect of 4-OH-Guanabenz on plasma glucose (**a**), triglyceride (**b**), and total cholesterol (**c**) levels in male Wistar rats fed with a high-fat diet. Means ± SD, n = 6. Comparisons were performed by one-way ANOVA, Tukey post hoc test. *p < 0.05, ***p < 0.001 significant *vs*. control rats fed standard diet; ^*p* < 0.05, ^^^*p* < 0.001 significant *vs*. control rats fed high-fat diet (diet-induced obesity control group)

The animals fed high-fat feed (control with obesity) had elevated levels of glucose and triglycerides in their blood. On the other hand, in animals treated with the tested compound at both doses, significantly lower blood glucose levels were observed (*F*_3,20_ = 39.98,* p* < 0.0001, one-way ANOVA) compared to a group of obese animals treated with water (Fig. 7a).

4-OH-Guanabenz, at a dose of 5 mg/kg b.w., significantly decreased plasma triglyceride levels in rats fed a high-fat diet, compared to the level of these parameters in the obese control group (F_3,20_ = 3.702, *p* = 0.0287, one-way ANOVA) (Fig. 7b).

The highest plasma level of total cholesterol was found in the group fed high-fat feed and simultaneously treated with 4-OH-Guanabenz at a dose of 5 mg/kg b.w. (*F*_3,20_ = 3.764, *p* = 0.0272, one-way ANOVA) (Fig. 7c).

### Influence on core temperature of obese rats

**Fig. 8:**
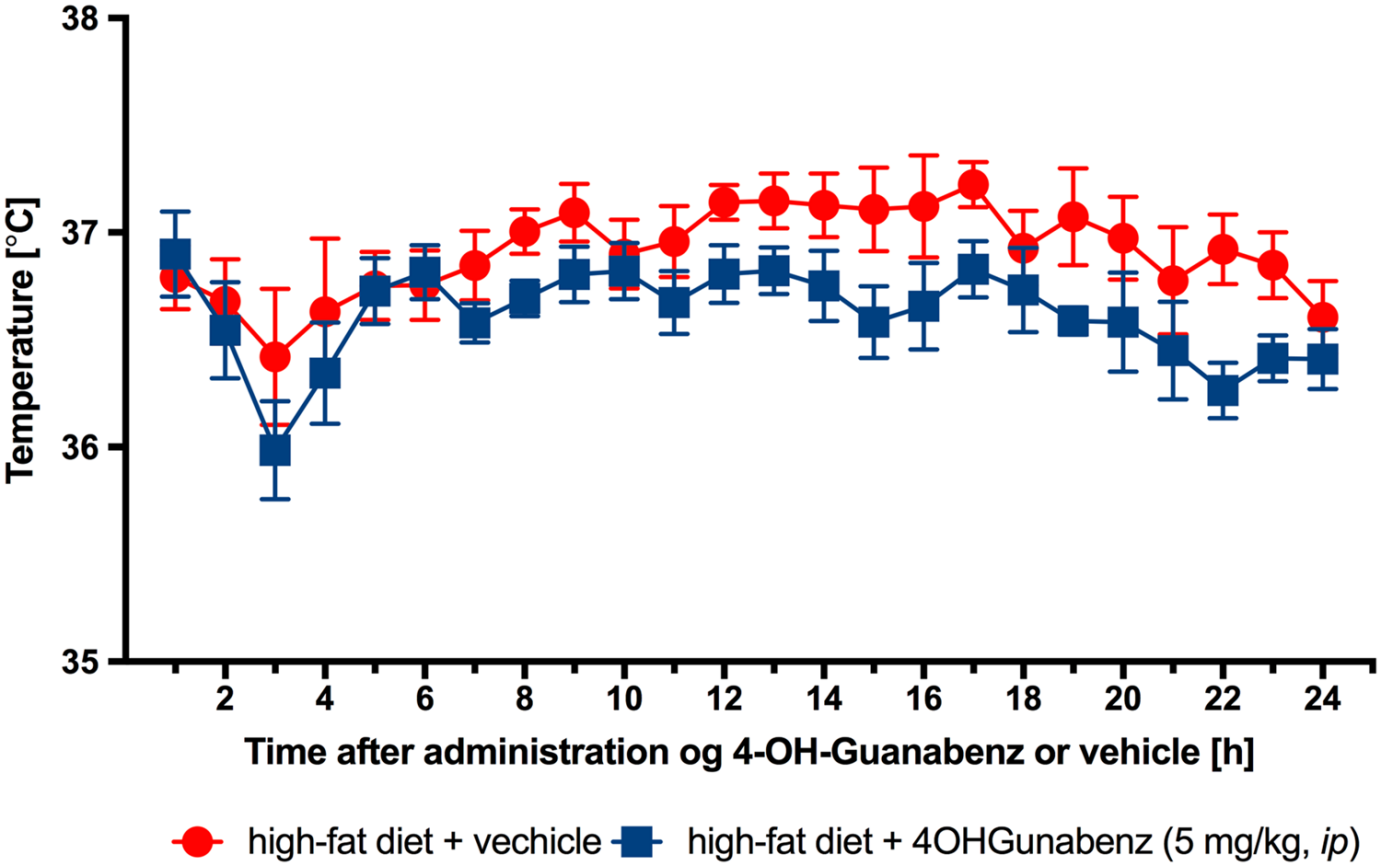
The effect of 4-OH-Guanabenz on the core temperature in male Wistar rats fed with a high-fat diet. Mean ± SEM, *n* = 6. Multiple comparisons were performed by two-way ANOVA for repeated measures

No significant effects on core temperature were noted in animals treated with 4-OH-Guanabenz at a dose of 5 mg/kg b.w. and consuming high-fat feed *vs* temperature noted in obese control rats (Fig. 8).

### Influence on body weight in rats fed a standard diet

No significant effects on body weight were noted in animals treated with 4-OH-Guanabenz at a dose of 5 mg/kg b.w. and eating standard feed (a trend towards a lower weight gain was observed) (Figure S1 Supplementary data).

### Influence on blood pressure

In rats sleeping under the influence of thiopental, blood pressure was not significantly different after administration of a single dose of 4-OH-Guanabenz (2 or 5 mg/kg b.w.) or water (Figure S2a or S2b supplementary data). Only after 4-OH-Guanabenz administration at a dose of 10 mg/kg b.w., a significant difference in diastolic pressure compared to the pressure determined in the control group was determined, but only in the 10th minute after administration (short-term increase) (Figure S2c Supplementary data).

**Fig. 9:**
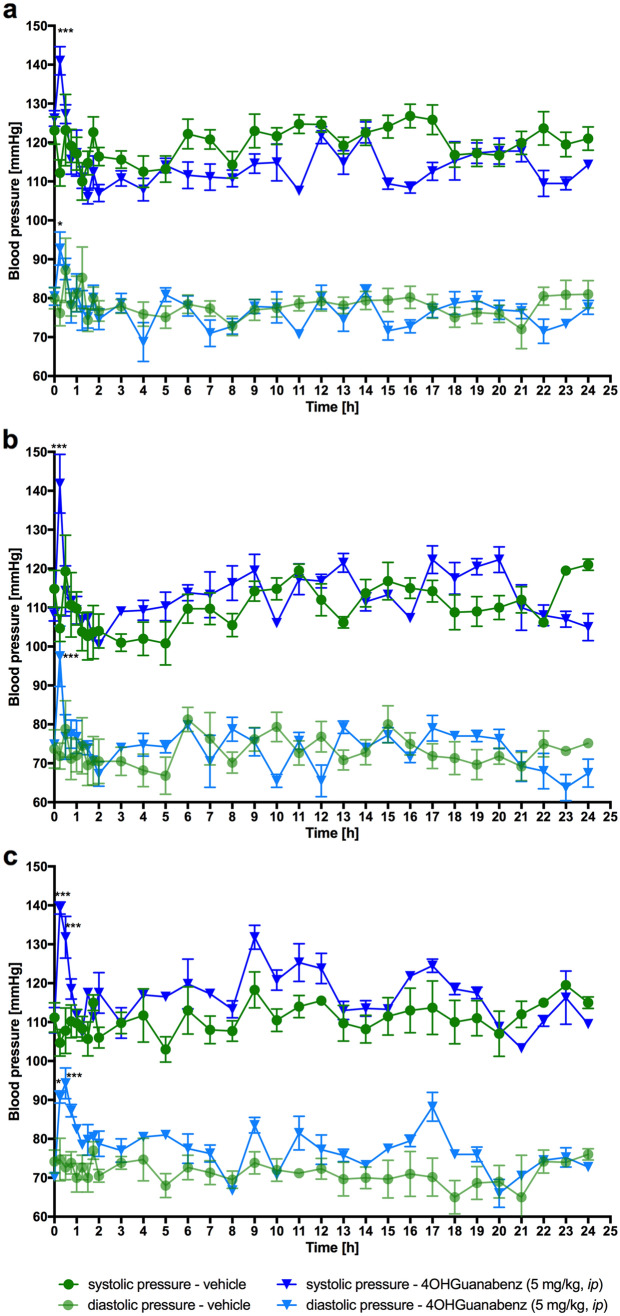
The effect of 4-OH-Guanabenz on blood pressure of normotensive rats (telemetric method). The changes of blood pressure during 24 h after the first (**a**), seventh (**b**), and fourteen (**c**) times administration. Mean ± SEM; *n* = 6. Multiple comparisons were performed by two-way ANOVA for repeated measures, Bonferroni post hoc test. *p < 0.05, ***p < 0.001 significant *vs*. control rats

In rats with normal activity (telemetry), both systolic and diastolic blood pressure were significantly higher in the group treated with 4-OH-Guanabenz (5 mg/kg b.w.) *vs* in the control group, but only in the first 15 or 15 and 30 min, after the first, seventh and fourteenth administration (Fig. 9).

### Pharmacokinetic analysis

The pharmacokinetic parameters for Guanabenz and 4-OH-Guanabenz calculated using the non-compartmental approach are given in Tables 4, 5, 6.

**Table 4:** Pharmacokinetic parameters for Guanabenz and 4-OH-Guanabenz after intraperitoneal administration to rats at a dose of 0.3 mg/kg and 0.4 mg/kg, respectively

Parameters	Guanabenz	4-OH-Guanabenz
AUC_0→t_ [ng ⋅ min/mL]	6449.5	5173.2
MRT [min]	117.5	95.2
t_0.5_ [min]	82.5	68.6
C_max_ [ng/mL]	95.1	100.5
t_max_ [min]	30	15
V_d_/F [mL/kg]	1608	1749
Cl/F [mL/min/kg]	13.5	17.7

*AUC* area under the curve, *MRT* mean residence time, *t*_*0.5*_ terminal half-life, *C*_*max*_ maximum concentration, *t*_*max*_ time to reach the maximum concentration, *V*_*d*_*/F* volume of distribution normalized on bioavailability, *Cl/F* clearance normalized on bioavailability

**Table 5:** Distribution in brain for Guanabenz and 4-OH-Guanabenz after intraperitoneal administration to rats at a dose of 0.3 mg/kg and 0.4 mg/kg, respectively

Parameters	Guanabenz	4-OH-Guanabenz
AUC_0→t_ [ng ⋅ min/g]	25,179	2573
*t*_0.5_ [min]	116.1	112.7
*C*_max_ [ng/g]	183.2	64.5
*t*_max_ [min]	60	15

*AUC* area under the curve, *t*_*0.5*_ terminal half-life, *C*_*max*_ maximum concentration in brain, *t*_*max*_ time to reach C_max_

**Table 6:** Partition coefficient between brain and plasma for Guanabenz and 4-OH-Guanabenz after intraperitoneal administration in rats at a dose of 0.3 mg/kg and 0.4 mg/kg, respectively

AUC_brain/plasma_	
Guanabenz	4-OH-Guanabenz
3.90	0.50

The pharmacokinetic results show that the absorption of Guanabenz and 4-OH-Guanabenz after *ip* administration was rather slow, with the peak concentration occurring at 30 min and 15 min, respectively. Following *ip* administration, guanabenz and 4-OH-Guanabenz were eliminated relatively quickly with the terminal half-lives of approximately 83 min and 69 min, respectively. The compounds showed a small volume of distribution—1.6 L/kg (Guanabenz) and 1.7 L/kg (4-OH-Guanabenz) that indicated their ability to penetrate deep compartments. Guanabenz was characterized by high brain permeability with a brain-to-plasma ratio of 3.9, while the 4-OH-Guanabenz permeability was markedly smaller—0.5. The penetration of guanabenz into the brain was slow and occurred within 60 min (*t*_max_), with a maximum concentration of 183.2 ng/g, while the penetration of 4-OH-Guanabenz into the brain was fast (*t*_max_ = 15 min), however, with a lower maximum concentration of 64.5 ng/g.

**Fig. 10:**
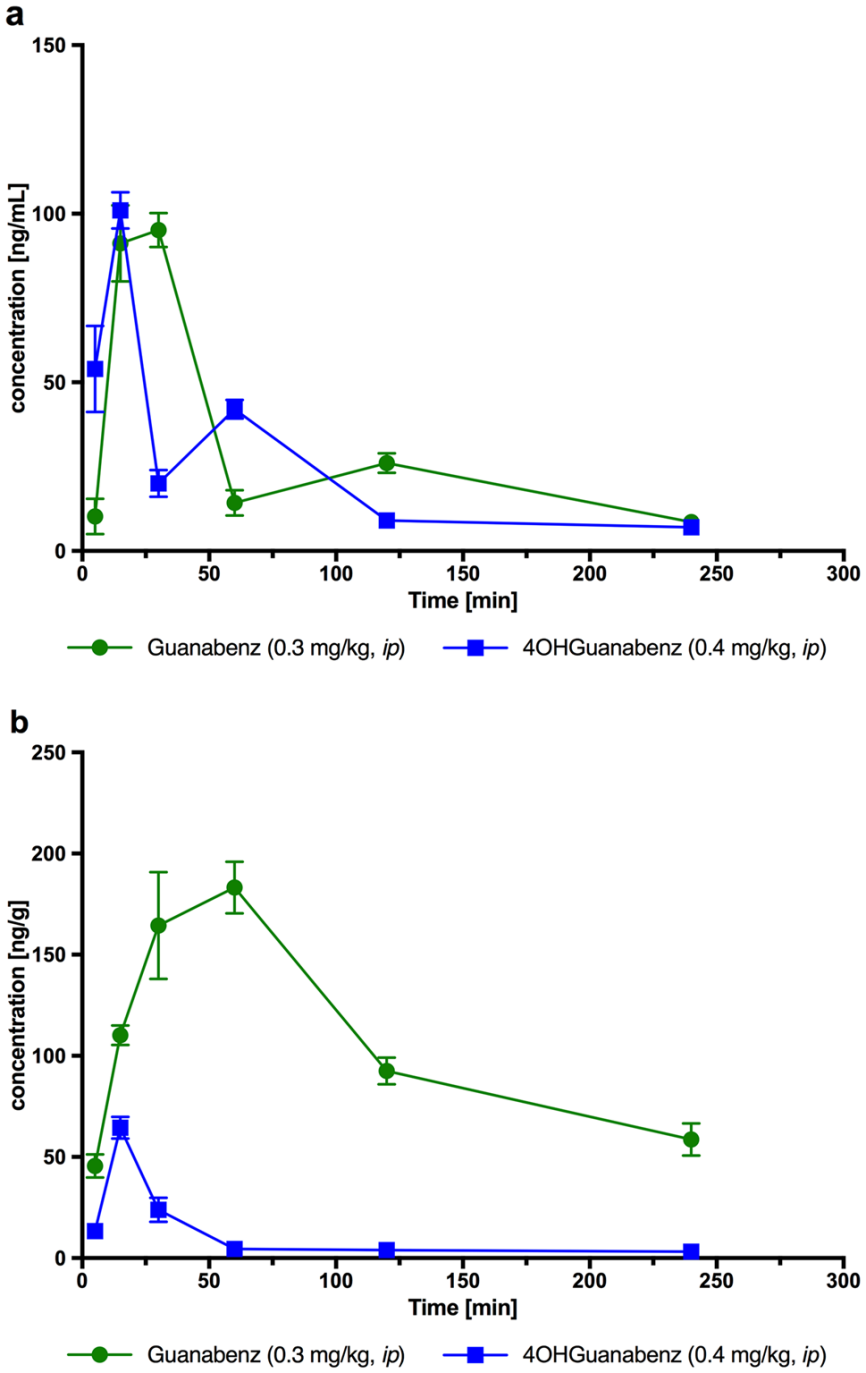
Concentration–time profiles in plasma (**a**) or brain (**b**) after administration of guanabenz (0.3 mg/kg) or 4-OH-Guanabenz (0.4 mg/kg) in rats. Mean ± SEM, *n* = 4

The plots of mean plasma and brain concentrations versus time profile for Guanabenz and 4-OH-Guanabenz after *ip* administration are shown in Fig. 10a and b.

## Discussion

The key findings of the present study are as follows: (i) 4-OH-Guanabenz causes a significant decrease in body weight of animals with developed obesity, (ii) a significant decrease in blood glucose and triglycerides levels, (iii) it is a partial agonist of the α_2A_-adrenoceptor and may act at TAAR_1_, (iv) it does not significantly affect body core temperature, (v) it does not significantly affect locomotor activity, and (vi) its effect on blood pressure does not appear to be significant.

In our previous studies, we have shown that guanabenz supports weight loss and ameliorates some metabolic disorders in obese animals [18]. In this study, we decided to test its metabolite in a similar manner. Given the limited information on 4-OH-Guanabenz in the literature, we started our investigation by determining its affinity and intrinsic activity toward α-adrenoceptors. Computer simulations have also shown other potential mechanisms of action, of which TAAR_1_ and serotonin 5-HT_2C_ receptors are particularly interesting for the treatment of obesity and metabolic syndrome. The literature reports on other α_2A_-adrenoceptor ligands, which simultaneously display an affinity for the serotonin 5-HT_2C_ receptor and TAAR_1_ [37]. The above characteristics make these compounds particularly interesting in terms of their influence on obesity, body weight, and selected metabolic parameters.

We found that, similarly to guanabenz, 4-OH-Guanabenz reduces body weight and caloric intake, and compensates for selected metabolic disorders in obese rats in a dose-dependent manner. Interestingly, it exhibits a different receptor profile to guanabenz. Table 7 summarizes the comparison of the pharmacological effects of guanabenz and 4-OH-Guanabenz. Our studies have shown that 4-OH-Guanabenz is a partial agonist of the α_2A_-adrenoceptor and does not bind to the α_1_-adrenoceptor, in contrast to guanabenz, which is an α_2A_-adrenergic agonist and blocks α_1_-adrenoceptors [38].

**Table 7:** Comparison of the pharmacological effects of guanabenz and 4OH-Guanabenz (the administered dose—5 mg/kg b.w., i.p.)

Parameter	Guanabenz	4OH-Guanabenz
Affinity towards α_1_-adrenoceptors	+	–
Affinity towards α_2_-adrenoceptors	+	+
Intrinsic activity at α_2A_-adrenoceptors	Full agonist	Partial agonist
Intrinsic activity at TAAR_1_	Agonist [48]	+
Body weight of obese animals	↓ [17]	↓
Amount of calories consumed	↓ [17]	↓
Spontaneous activity	↓ [17]	–
Plasma glucose level	↓ [17]	↓
Plasma triglyceride level	↓ [17]	↓
Plasma cholesterol level	↑ [17]	↑
Core temperature	↓ [17]	–
Blood pressure after one administration	Change [17]	–
Penetration into the brain	Good	Poor

+ Action, – No action, ↓ Reduction, ↑ Gain

It is generally accepted that α_1_-blockers, administered chronically, have a beneficial effect on lipid and carbohydrate profiles [35, 39, 40]. Therefore, the ability to block α_1_-adrenoceptors by guanabenz in obesity appears to be beneficial. However, blocking α_1_-adrenoceptors may also cause side effects such as sedation, vasodilation, and a drop in blood pressure. 4-OH-Guanabenz has no α_1_-blocking activity, but it also reduces body weight and balances some metabolic parameters. This may be related to its activity of the partial reduction of the response mediated through α_2A_-adrenoceptor (due to the partial agonistic activity at α_2A_-adrenoceptor). Recently published data revealed the ability of α_2A_-adrenoceptor antagonists to induce weight loss in animal models of obesity [7, 9, 32]. Our study, for the first time, demonstrated that partial stimulation of α_2A_-adrenoceptor, which also subtly reduces the receptor response, may be sufficient to produce beneficial effects on body weight and some metabolic disturbances in obese animals. Obviously, we do not exclude the participation of additional targets, and we are already planning extended research in this direction for this active and extremely interesting compound.

4-OH-Guanabenz significantly reduced the elevated plasma glucose level in the obesity state induced by diet. During physiological conditions, catecholamines that act on postsynaptic α_2A_-adrenoceptors located on pancreatic islet β-cells inhibit insulin secretion and consequently increase blood glucose levels [41]. Thus, reduction of the α_2A_-adrenoceptor activity may facilitate insulin secretion from pancreatic islets and, as a result, reduce elevated glucose levels [42]. In our previous study, we showed that, apart from reduction in body weight and selected metabolic disorders, guanabenz also normalized the glucose levels raised by obesity in rats. This was an unexpected observation, considering that guanabenz is an α_2A_-adrenoceptor agonist. Of note, these findings were confirmed also by the other studies [19, 43]. The possibility that, in addition to diminished activity of the α_2A_-adrenoceptor receptor, other mechanisms of action of these compounds may account for reduced plasma glucose levels and these are clearly evident.

The observed reduction in triglyceride and glucose levels after the administration of 4-OH-Guanabenz over a 25-day period may suggest that its prolonged use could lead to the reduction of body weight in the test animals. This could be a result of a balanced lipid and carbohydrate economy, which is often an important first step toward weight loss.

One of the more recent trends in type 2 diabetes mellitus therapies has focused on incretin hormones [44]. Stable incretin mimetics have been found to be effective in improving glycaemic control in diabetes, with the added benefit of modest weight loss [45]. TAAR_1_ is a receptor coupled with G proteins. It is expressed, inter alia, in the stomach, duodenum, and pancreatic β-cells [46, 47]. Activation of TAAR_1_ has beneficial effects on glucose control and body weight in animal models of type 2 diabetes and obesity by incretin-like effects [46]. An additional beneficial effect is the reduction of monoaminergic signaling in the brain, via down-regulation of dopamine reward circuits mediated by TAAR_1_ [47, 48]. Previous studies in mice showed that guanabenz caused an increase in blood glucagon-like peptide 1 (GLP-1) and insulin levels, a decrease in blood glucose level, and an elevation of leptin level along with a reduction in food intake [42]. In addition, guanabenz has been shown to be an agonist of TAAR_1_ [49]. Therefore, a significant limitation of our research is the lack of data evaluating insulin and leptin levels in plasma collected from animals after the administration of 4-OH-Guanabez. However, we consider the present study preliminary, and we are already planning to extend it with important elements that confirm the mechanisms of action of 4-OH-Guanabenz, specifically with the possibility of acting by TAAR_1_. Our results show for the first time that 4-OH-Guanabenz may act in TAAR_1_; therefore, some of its beneficial effects, i.e., reducing food intake, reducing weight in obese animals, and lowering the level of glucose and triglycerides in the plasma, may be related to this mechanism. This, of course, requires further research.

The results of pharmacokinetic studies showed that 4-OH-Guanabenz exhibits drug-like properties. 4-OH-Guanabenz showed rapid and poor penetration into the brain, indicating that its action through central mechanisms appears to be less important than through peripheral mechanisms (α_2A_-adrenoceptors and TAAR_1_ in pancreatic islets, adipose tissue, or gastrointestinal track). Our in silico studies have shown that this compound may be a ligand for the serotonin 5-HT_2C_ receptor, which may be important in its anorectic action (if it is its agonist). The serotonin 5-HT_2C_ receptor plays a very important role in the regulation of the state of satiety, but it is currently believed that this regulation is carried out centrally [50]. In any case, the effect of 4-OH-Guanabenz on the serotonin 5-HT_2C_ receptor requires further research.

Changes in spontaneous locomotor activity could be a possible indicator of the side effects of a tested compound [51]. Animals that feel unwell under the influence of the administered compound (for various reasons, for example, hypotension, toxic effects, pain, etc.) are often sedated. Sedation may also be a factor in biasing the results of tests for compounds sought for obesity treatment, because when animals are overly sedated, they may consume less food compared to animals in a physiological state, which can contribute to weight reduction. Previous research shows that guanabenz has a significant undesirable effect in the form of sedation [18]. In the present study, 4-OH-Guanabenz did not significantly affect spontaneous activity. The lack of a sedative effect may be related to its weaker central activity, which is advantageous from the point of view of not being able to cause central side effects. Additionally, physiological locomotor activity confirms that the animals do not feel unwell after administration of 4-OH-Guanabenz, and indirectly indicates that it is well tolerated by the body.

Our studies did not indicate significant differences in the effect on core body temperature between the control and 4-OH-Guanabenz groups. This is another difference between 4-OH-Guanabenz and guanabenz, since the latter significantly lowered the core body temperature of rats, as we showed in our previous study [18].

Next, the present work also showed a slight influence of 4-OH-Guanabenz on blood pressure. It should be highlighted that guanabenz was originally approved for the treatment of hypertension [12]. In patients with metabolic syndrome, where in addition to obesity there are other disorders such as hypertension, the effect of lowering blood pressure is obviously beneficial. However, the lack of such an effect can also be considered as an advantage, as patients are often treated with additional cardiovascular drugs. Therefore, the lack of a significant effect on the blood pressure of 4-OH-Gunabanez would be associated with the lack of adverse interactions in this regard.

In summary, our research clearly shows for the first time that 4-OH-Guanabenz is able to effectively reduce the body weight of obese animals and compensate for disturbances in selected metabolic parameters. 4-OH-Guanabenz is a ligand of α_2A_-adrenoceptor and TAAR_1_, acting mainly peripherally. However, the exact sequence of molecular events in the organism, connecting the influence of 4-OH-Guanabenz on α_2A_-adrenoceptor and TAAR_1_ with weight reduction and improvement of metabolic disturbances, remains an open question and requires further studies. Undoubtedly, the fact that 4-OH-Guanabenz is a metabolite of a well-known drug is considerably important and beneficial from an economic point of view and towards its further development as a drug candidate.

### Supplementary Information

Below is the link to the electronic supplementary material.

Supplementary file1 (DOCX 1034 KB)

Supplementary file2 (XLSX 75 KB)

Supplementary file3 Figure S1. The effect of 4-OH-Guanabenz on body weight in male Wistar rats fed with standard diet. The changes in body weight in control (standard diet) and in Wistar rats fed standard diet treated for 25 days with the tested compound. Mean ± SEM, n=4. Multiple comparisons were performed by two-way ANOVA with repeated measure (TIFF 336 KB)

Supplementary file4 Figure S2. The effect of 4-OH-Guanabenz on blood pressure of normotensive rats after a single administration. The changes in systolic and diastolic blood pressure after single, ip administration of the test compound at doses of 2 (a) or 5 (b) or 10 (c) mg/kg b.w. to rats fed standard diet. Mean ± SEM, n=6. Multiple comparisons were performed by two-way ANOVA with repeated measures, Bonferroni post-hoc test. *p<0.05 significant vs. control rats (TIFF 502 KB)

## Data Availability

The data presented in this study are available in a Supplementary materials.

## References

[1] Jocken J, Blaak E (2008). Catecholamine-induced lipolysis in adipose tissue and skeletal muscle in obesity. Physiol Behav.

[2] Pujol E, Rodriguez-Cuenca S, Frontera M, Justo R, Llado I, Kraemer FB (2003). Gender- and site-related effects on lipolytic capacity of rat white adipose tissue. Cell Mol Life Sci.

[3] Langin D (2006). Adipose tissue lipolysis as a metabolic pathway to define pharmacological strategies against obesity and the metabolic syndrome. Pharmacol Res.

[4] Arner P (1999). Catecholamine-induced lipolysis in obesity. Int J Obes.

[5] Lafontan M, Berlan M, Galitzky J, Montastruc J-L (1992). Alpha-2 adrenoceptors in lipolysis: α2 antagonists and lipid-mobilizing strategies. Am J Clin Nutr.

[6] Galitzky J, Vermorel M, Lafontan M, Montastruc P, Berlan M (1991). Thermogenetic and lipolytic effect of yohimbine in the dog. Br J Pharmacol.

[7] Dudek M, Knutelska J, Bednarski M, Nowiński L, Zygmunt M, Mordyl B (2015). A comparison of the anorectic effect and safety of the alpha2-adrenoceptor ligands guanfacine and yohimbine in rats with diet-induced obesity. PLoS ONE.

[8] Janhunen SK, van der Zwaal EM, la Fleur SE, Adan RAH (2011). Inverse agonism at α2A adrenoceptors augments the hypophagic effect of sibutramine in rats. Obesity.

[9] Janhunen SK, la Fleur SE, Adan RA (2013). Blocking alpha2A adrenoceptors, but not dopamine receptors, augments bupropion-induced hypophagia in rats. Obesity.

[10] Philipp M, Brede M, Hein L (2002). Physiological significance of a2-adrenergic receptor subtype diversity: one receptor is not enough. Am J Physiol Regul Integr Comp Physiol.

[11] Tank J, Heusser K, Diedrich A, Brychta R, Luft F, Jordan J (2007). Yohimbine attenuates baroreflex-mediated bradykardia in humans. Hypertension.

[12] Holmes B, Brogden RN, Heel RC, Speight TM, Guanabenz AGS (1983). A review of its pharmacodynamic properties and therapeutic efficacy in hypertension. Drugs.

[13] Sun X, Aimé P, Dai D (2018). Guanabenz promotes neuronal survival via enhancement of ATF4 and parkin expression in models of Parkinson disease. Exp Neurol.

[14] Vaccaro A, Patten SA, Aggad D (2013). Pharmacological reduction of ER stress protects against TDP-43 neuronal toxicity in vivo. Neurobiol Dis.

[15] Singh A, Gupta P, Tiwari S, Mishra A, Singh S (2022). Guanabenz mitigates the neuropathological alterations and cell death in Alzheimer's disease. Cell Tissue Res.

[16] Tanaka M, Inoue Y, Imai T, Tanida N, Takahashi K, Hara H (2021). Guanabenz and clonidine, alpha2-adrenergic receptor agonists inhibit choroidal neovascularization. Curr Neurovasc Res.

[17] Xie W, Xie J, Vince R, More SS (2020). Guanabenz attenuates acetaminophen-induced liver toxicity and synergizes analgesia in mice. Chem Res Toxicol.

[18] Kotańska M, Knutelska J, Nicosia N, Mika K, Szafarz M (2022). Guanabenz—an old drug with a potential to decrease obesity. Naunyn Schmiedebergs Arch Pharmacol.

[19] Yoshino S, Iwasaki Y, Matsumoto S, Satoh T, Ozawa A, Yamada E (2020). Administration of small-molecule guanabenz acetate attenuates fatty liver and hyperglycemia associated with obesity. Sci Rep.

[20] Orhan H (2015). Extrahepatic targets and cellular reactivity of drug metabolites. Curr Med Chem.

[21] Obach RS (2013). Pharmacologically active drug metabolites: impact on drug discovery and pharmacotherapy. Pharmacol Rev.

[22] Fluck ER, Homon CA, Knowles JA, Ruelius HW (1983). Differential binding of guanabenz and its metabolites to cerebral aa-receptors: the basis for a radioligand assay specific for the drug. Drug Dev Res.

[23] Daina A, Michielin O, Zoete V (2019). SwissTargetPrediction: updated data and new features for efficient prediction of protein targets of small molecules. Nucleic Acids Res.

[24] Sydow D, Wichmann M, Rodríguez-Guerra J, Goldmann D, Landrum G, Volkamer A. TeachOpenCADD-KNIME: A Teaching Platform for Computer-Aided Drug Design Using KNIME Workflows. 2019. 10.5281/zenodo.3475086 10.1021/acs.jcim.9b00662 31612715

[25] Marvin was used for drawing, displaying and characterizing chemical structures, 21.1.0, 2021, ChemAxon (http://www.chemaxon.com)

[26] Schrödinger Release 2021–4: Protein Preparation Wizard; Epik, Schrödinger, LLC, New York, NY, 2021; Impact, Schrödinger, LLC, New York, NY; LigPrep , Schrödinger, LLC, New York, NY; Prime, Schrödinger, LLC, New York, NY, 2021.Maestro, Schrödinger, LLC, New York.

[27] Sastry GM, Adzhigirey M, Day T, Annabhimoju R, Sherman W (2013). Protein and ligand preparation: Parameters, protocols, and influence on virtual screening enrichments. J Comput Aid Mol Des.

[28] Jendele L, Krivak R, Skoda P, Novotny M, Hoksza D (2019). PrankWeb: a web server for ligand binding site prediction and visualization. Nucleic Acids Res.

[29] Halgren TA, Murphy RB, Friesner RA, Beard HS, Frye LL, Pollard WT (2004). Glide: a new approach for rapid, accurate docking and scoring 2 Enrichment factors in database screening. J Med Chem.

[30] Marcinkowska M, Kotańska M, Zagórska A, Śniecikowska J, Kubacka M, Siwek A (2018). Synthesis and biological evaluation of N-arylpiperazine derivatives of 4,4-dimethylisoquinoline-1,3(2H,4H)-dione as potential antiplatelet agents. J Enzyme Inhib Med Chem.

[31] Dudek M, Marcinkowska M, Bucki A, Olczyk A, Kołaczkowski M (2015). Idalopirdine—a small molecule antagonist of 5-HT6 with therapeutic potential against obesity. Metab Brain Dis.

[32] Dudek M, Knutelska J, Bednarski M, Nowiński L, Zygmunt M, Kazek G (2016). Pyrrolidin-2-one derivatives may reduce body weight in rats with diet-induced obesity. Eur J Pharmacol.

[33] Dudek M, Kuder K, Kołaczkowski M, Olczyk A, Żmudzka E, Rak A (2016). H3 histamine receptor antagonist pitolisant reverses some subchronic disturbances induced by olanzapine in mice. Metab Brain Dis.

[34] Kotańska M, Śniecikowska J, Jastrzębska-Więsek M, Kołaczkowski M, Pytka K (2017). Metabolic and cardiovascular benefits and risks of EMD386088-A 5-HT6 receptor partial agonist and dopamine transporter inhibitor. Front Neurosci.

[35] Kotańska M, Kulig K, Marcinkowska M, Bednarski M, Malawska K, Zaręba P (2018). Metabolic benefits of 1-(3-(4-(o-tolyl)piperazin-1-yl)propyl)pyrrolidin-2-one: a non-selective α-adrenoceptor antagonist. J Endocrinol Invest.

[36] Francesconi V, Cichero E, Kanov EV, Laurini E, Pricl S, Gainetdinov RR (2020). Novel 1-amidino-4-phenylpiperazines as potent agonists at human taar1 receptor: Rational design, synthesis, biological evaluation and molecular docking studies. Pharmaceuticals.

[37] Simmler LD, Rickli A, Schramm Y, Hoener MC, Liechti ME (2014). Pharmacological profiles of aminoindanes, piperazines, and pipradrol de-rivatives. Biochem Pharmacol.

[38] Takeuchi K, Kogure M, Hashimoto T (1987). Comparison of agonistic and antagonistic action of guanabenz and guanfacine on α1 and α2-adrenoreceptors in isolated smooth muscles. Japan J Pharmacol.

[39] Deshaies Y (1993). Postprandial plasma triacylglycerols under α1-adrenergic blockade. Am J Physiol Endocrinol Metab.

[40] Pessina AC, Ciccariello L, Perrone F, Stoico V, Gussoni G, Scotti A (2006). Clinical efficacy and tolerability of alpha-blocker doxazosin as add-on therapy in patients with hypertension and impaired glucose metabolism. Nutr Metab Cardiovasc Dis.

[41] Adefurin A, Darghosian L, Okafor C, Kawai V, Li C, Shah A (2016). Alpha2A adrenergic receptor genetic variation contributes to hyperglycemia after myocardial infarction. Int J Cardiol.

[42] Rosengren AH, Braun M, Mahdi T, Andersson SA, Travers ME, Shigeto M (2012). Reduced insulin exocytosis in human pancreatic β-cells with gene variants linked to type 2 diabetes. Diabetes.

[43] Ye H, Charpin-El Hamri G, Zwicky K, Christen M, Folcher M, Fussenegger M (2013). Pharmaceutically controlled designer circuit for the treatment of the metabolic syndrome. Proc Natl Acad Sci U S A.

[44] Nauck MA, Quast DR, Wefers J, Pfeiffer AFH (2021). The evolving story of incretins (GIP and GLP-1) in metabolic and cardiovascular disease: a pathophysiological update. Diabetes Obes Metab.

[45] Goldenber R (2014). Insulin plus incretin agent combination therapy in type 2 diabetes: a systematic review. Curr Med Res Opin.

[46] Raab S, Wang H, Uhles S, Cole N, Alvarez-Sanchez R, Künnecke B (2015). Incretin-like effects of small molecule trace amine-associated receptor 1 agonists. Mol Metab.

[47] Michael ES, Covic L, Kuliopulos A (2019). Trace amine-associated receptor 1 (TAAR1) promotes anti-diabetic signaling in insulin-secreting cells. J Biol Chem.

[48] Revel FG, Moreau J-L, Gainetdinov RR, Bradaia A, Sotnikova TD, Mory R (2011). TAAR1 activation modulates monoaminergic neurotransmis-sion, preventing hyperdopaminergic and hypoglutamatergic activity. Proc Natl Acad Sci.

[49] Hu LA, Zhou T, Ahn J, Wang S, Zhou J, Hu Y (2009). Human and mouse trace amine-associated receptor 1 have distinct pharmacology towards endogenous monoamines and imidazoline receptor ligands. Biochem J.

[50] Voigt J-P, Fink H (2015). Serotonin controlling feeding and satiety. Behav Brain Res.

[51] Lynch JJ, Castagné V, Moser PC, Mittelstadt SW (2011). Comparison of methods for the assessment of locomotor activity in rodent safety pharmacology studies. J Pharmacol Toxicol Methods.

