# Supplementary material for: Metabolic and cardiovascular benefits and risks of 4-hydroxy guanabenz hydrochloride: α2-adrenoceptor and trace amine-associated receptor 1 ligand
Source: Pharmacol Rep. 2023 Aug 25;75(5):1211–29. doi: 10.1007/s43440-023-00518-9 (PMC10539439; doi:10.1007/s43440-023-00518-9)

*Supplementary data:*

**NMR and UPLC-UV-MS spectra of tested compound.**

2-[(2,6-Dichloro-4-hydroxyphenyl)methylene]hydrazinecarboximidamide hydrochloride

**1HNMR** (500 MHz, METHANOL-d4)

(METHANOL-d4, 500 MHz) δ 8.35 (s, 1H), 6.90 (s, 2H), NH protons not detected


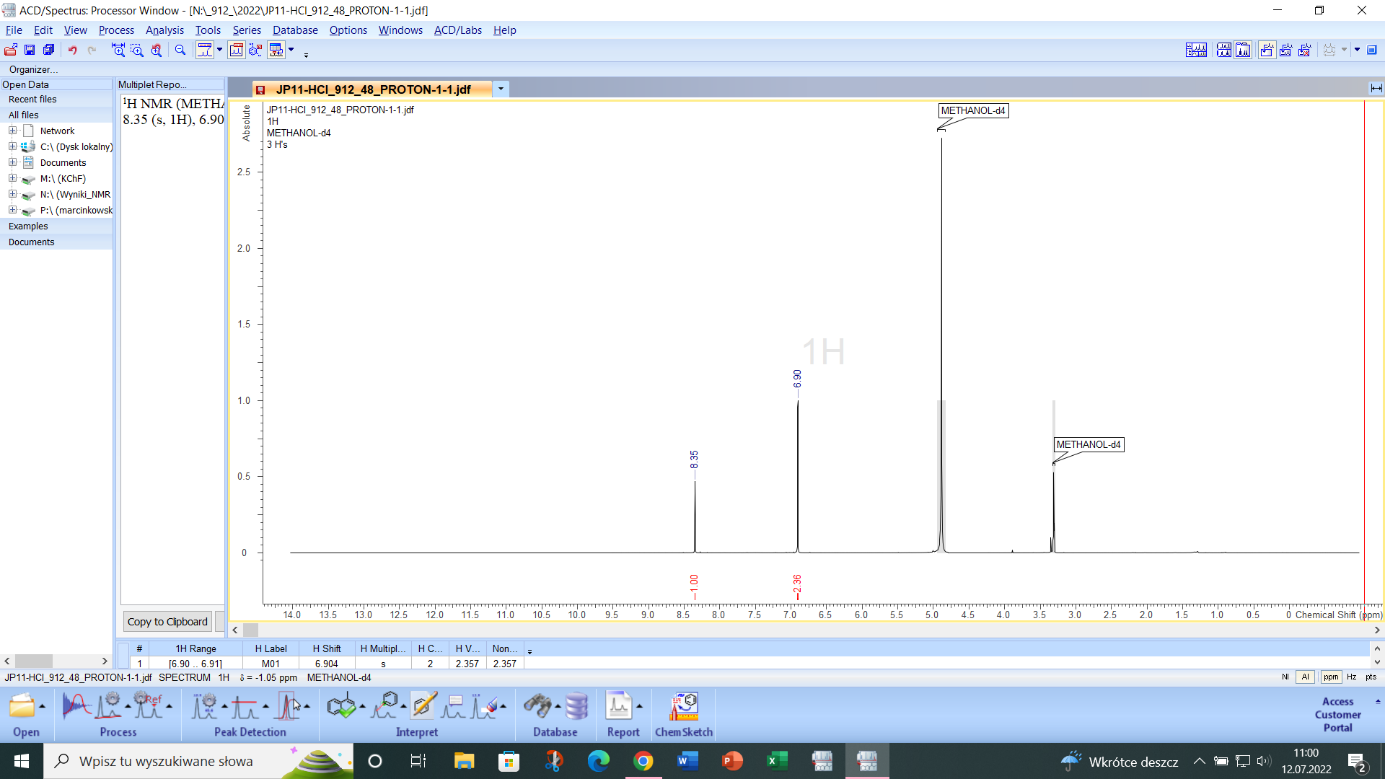


**13C NMR** (126 MHz, METHANOL-d4)


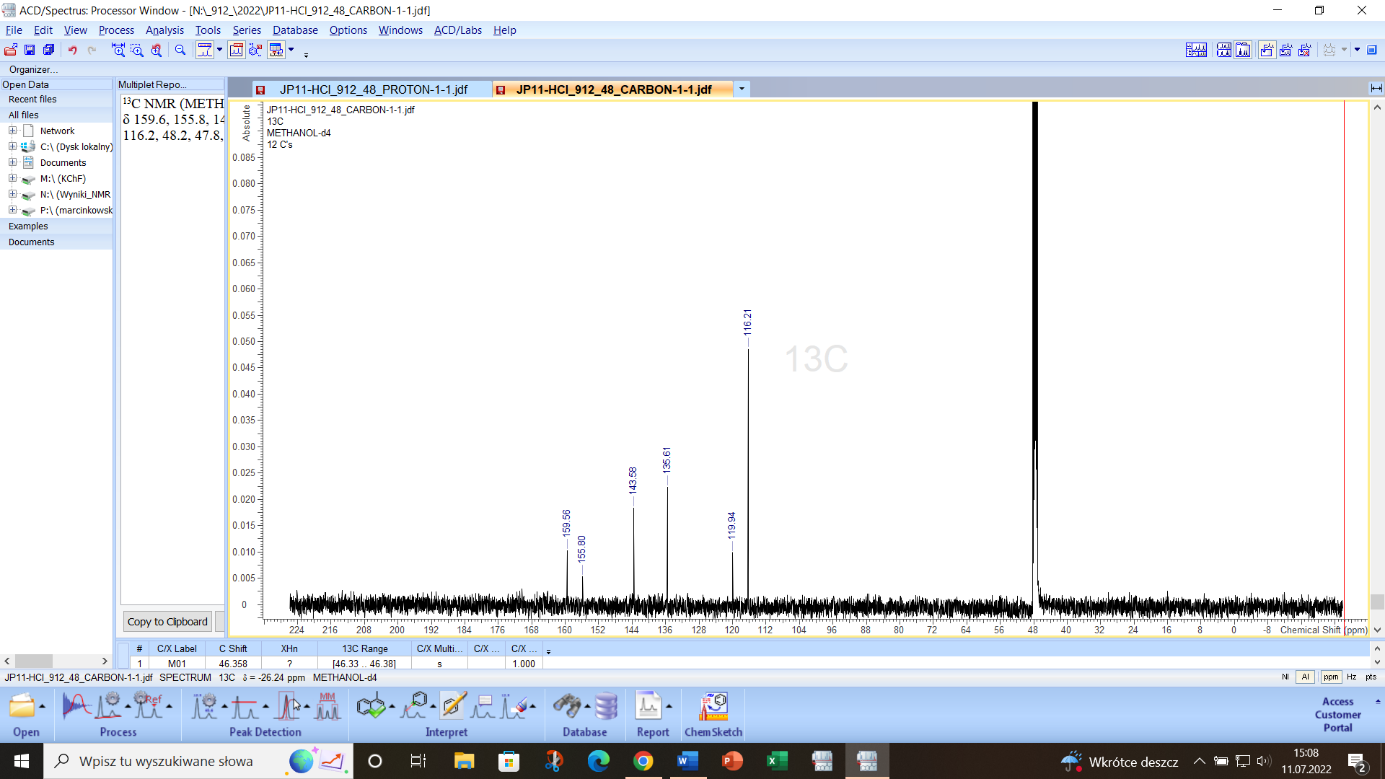


**UPLC-UV-MS** (200–700 nm), calcd for C_8_H_9_Cl_3_N_5_O: 247.01 (M+H+), found: 246.96 (M+H+).

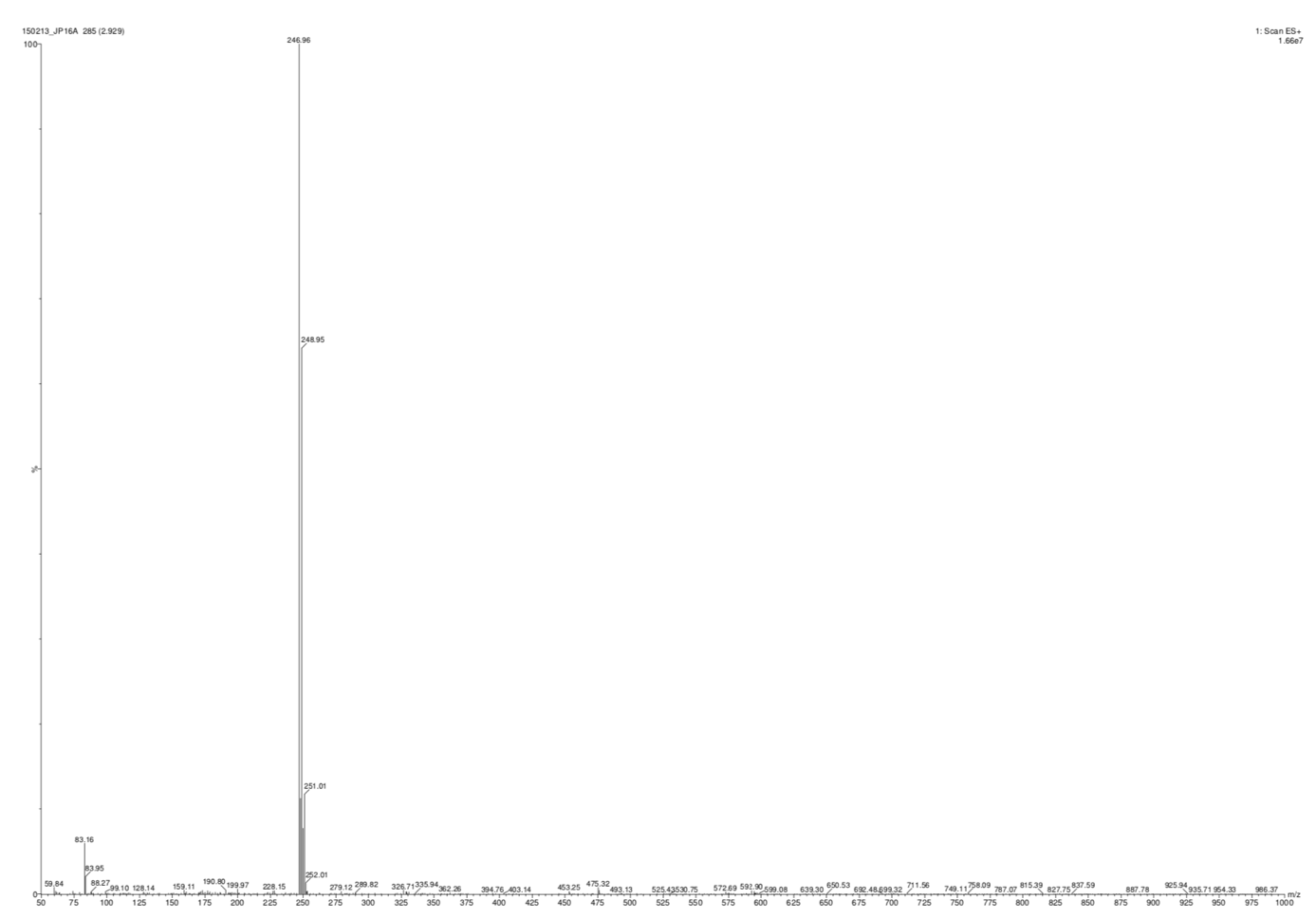

Supplement: Supplementary file 1 — Supplementary file1 (DOCX 1034 KB) [file 43440_2023_518_MOESM1_ESM.docx]
