# Supplementary figures and images for: Metabolic and cardiovascular benefits and risks of 4-hydroxy guanabenz hydrochloride: α2-adrenoceptor and trace amine-associated receptor 1 ligand
Source: Pharmacol Rep. 2023 Aug 25;75(5):1211–29. doi: 10.1007/s43440-023-00518-9 (PMC10539439; doi:10.1007/s43440-023-00518-9)

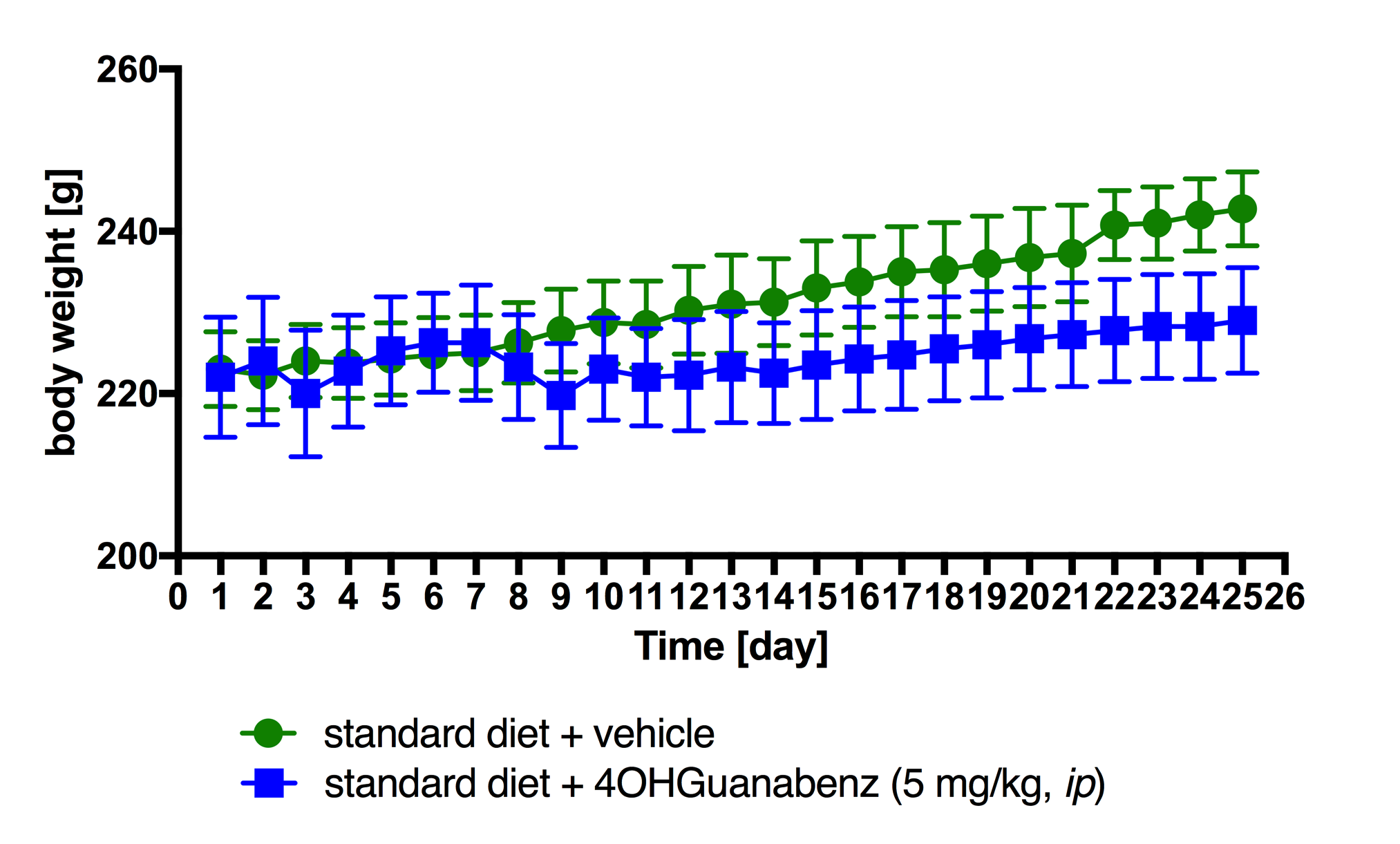

Supplement: Supplementary file 3 — Supplementary file3 Figure S1. The effect of 4-OH-Guanabenz on body weight in male Wistar rats fed with standard diet. The changes in body weight in control (standard diet) and in Wistar rats fed standard diet treated for 25 days with the tested compound. Mean ± SEM, n=4. Multiple comparisons were performed by two-way ANOVA with repeated measure (TIFF 336 KB) [file 43440_2023_518_MOESM3_ESM.tiff]

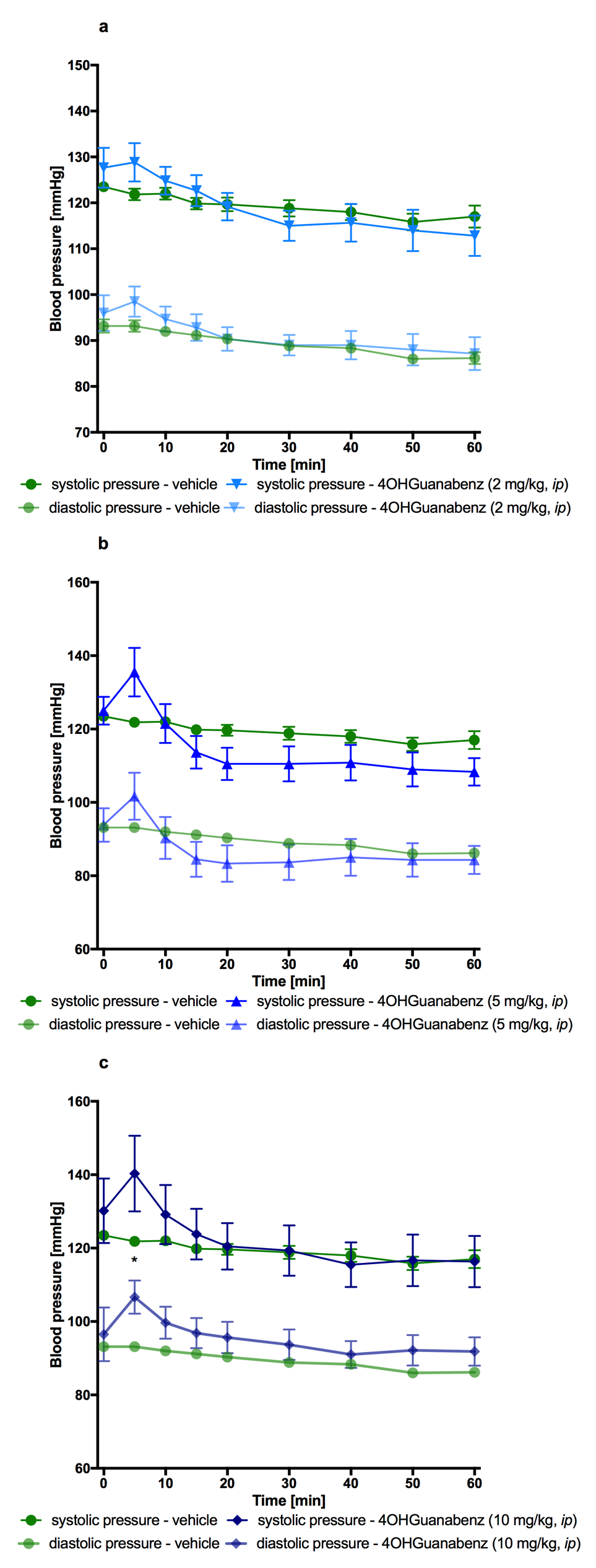

Supplement: Supplementary file 4 — Supplementary file4 Figure S2. The effect of 4-OH-Guanabenz on blood pressure of normotensive rats after a single administration. The changes in systolic and diastolic blood pressure after single, ip administration of the test compound at doses of 2 (a) or 5 (b) or 10 (c) mg/kg b.w. to rats fed standard diet. Mean ± SEM, n=6. Multiple comparisons were performed by two-way ANOVA with repeated measures, Bonferroni post-hoc test. *p<0.05 significant vs. control rats (TIFF 502 KB) [file 43440_2023_518_MOESM4_ESM.tiff]
